# A Bio-Inspired Physiological–Behavioral Fusion Method for Human-Factor Risk Assessment of Imported Unmanned Intelligent System Operators in Port-Security Scenarios

**DOI:** 10.3390/biomimetics11090675

**Published:** 2026-09-19

**Authors:** Nanfeng Zhang, Ying Dong, Xin Liao, Liuhua Zhang, Honggang Wang, Genjian Yang, Jinchao Xiao, Jingfeng Yang

**Affiliations:** 1 Guangdong Provincial Key Laboratory of Intelligent Port Security Inspection, Huangpu Customs District P.R.China, Guangzhou 510700, China; zhangnanfeng@xupt.edu.cn (N.Z.); xiaojinchao@sia.cn (J.X.); 2 China Electronic Product Reliability and Environment Test Research Institute, Guangzhou 511300, China; liaox@ceprei.org (X.L.); zhanglh@ceprei.org (L.Z.); yanggj@ceprei.org (G.Y.); 3 School of Communications and Information Engineering and School of Artificial Intelligence, Xi’an University of Posts and Telecommunications, Xi’an 710121, China; wanghonggang@xupt.edu.cn; 4 IoT Technology and Application R&D Center, Guangzhou Institute of Industrial Intelligence, Guangzhou 511458, China

**Keywords:** bio-inspired perception, unmanned intelligent systems, human-factor risk assessment, physiological–behavioral fusion, operator state recognition, attention mechanism, human–machine collaborative safety

## Abstract

Operator fatigue, stress, delayed response, and abnormal operation may compromise the safety of unmanned intelligent system operation in port-security scenarios. This study proposes a bio-inspired engineering architecture for physiological–behavioral fusion risk assessment of operators. The architecture is motivated by homeostatic regulation, coordinated physiological–behavioral responses, selective attention, and feedback concepts; it does not aim to reproduce a biological or neural mechanism. It combines operator-specific baseline calibration, physiological and behavioral feature representation, cross-modal coupling representation, modality-level adaptive weighting, and temporal risk-state classification to identify normal, fatigue, stress, and abnormal-operation states. Physiological information was acquired using a non-invasive smart-cushion sensing system and supplementary electrodermal-activity monitoring, while behavioral information was derived from operation-platform and task-event logs. The proposed method was evaluated under representative port-operation tasks using a participant-level data split and repeated computational training-and-evaluation runs. These repeated runs were used to characterize stochastic optimization stability and were not interpreted as independent participant experiments. The results indicate that the proposed method achieved improved overall classification performance, warning-related reliability, and repeated-run stability compared with single-modal, conventional machine-learning, direct-concatenation, and temporal-fusion baselines. The findings provide pilot evidence that physiological–behavioral fusion can support operational human-factor risk-state classification under the examined port-operation conditions. Larger cross-operator and cross-scenario studies are required to establish broader generalizability.

## 1. Introduction

### 1.1. Research Background and Motivation

With the rapid development of artificial intelligence, automatic control, the Internet of Things, and next-generation communication technologies, unmanned aerial, ground, and marine platforms have become increasingly important components of intelligent transportation and automated operational systems [1]. These systems are increasingly used in logistics, inspection, emergency response, and public-safety applications. In port-security scenarios, imported unmanned intelligent systems may be involved in entry inspection, remote operation, intelligent patrol, equipment supervision, and safety assessment. Although these systems possess increasing levels of autonomy, their safe operation still depends on human operators who perform task planning, process monitoring, abnormality judgment, remote intervention, and safety takeover. Previous studies on drone-operation safety, driver performance, operator training, remote ship operations, and automation failures in unmanned systems have shown that operational skills, workload, situational awareness, task performance, and trust in automation can substantially affect operational safety [2,3,4,5,6].

During continuous monitoring, remote control, multi-device coordination, alarm handling, and emergency response, operators may experience fatigue, stress, excessive cognitive workload, delayed responses, judgment deviations, or abnormal operations. These human-factor risks do not originate from equipment faults alone; they may also arise from changes in the operator’s internal physiological state and external operational performance. Physiological indicators such as heart rate, respiration, electrodermal activity, and eye movement have been integrated through multimodal learning to evaluate driver fatigue, distraction, and potentially unsafe states [7,8,9,10,11,12,13,14,15]. Behavioral features, including control frequency, response delay, operation-trajectory deviation, command interval, erroneous operations, and task-completion time, can directly reflect operational stability and task-execution performance [11,16,17,18].

However, neither physiological monitoring nor behavioral analysis alone is sufficient for reliable human-factor risk assessment in complex port-operation scenarios. Physiological signals are sensitive to individual differences, sensor noise, motion artifacts, and environmental disturbances, whereas behavioral abnormalities may become apparent only after a risk state has developed. Therefore, the joint use of physiological and behavioral information is important for improving the completeness, timeliness, and robustness of operator-state assessment.

Although multimodal fusion has been increasingly applied to fatigue detection, physiological-signal analysis, and human–machine interaction monitoring, existing methods often rely on direct feature concatenation or simple decision-level fusion [16,17,18,19,20]. Such approaches may not sufficiently characterize the dynamic relationship between physiological-state deviation and behavioral abnormality, especially under conditions involving sustained task load, sudden alarms, or complex task switching. Therefore, this study proposes a bio-inspired physiological–behavioral fusion method for human-factor risk assessment of imported unmanned intelligent system operators in port-security scenarios. The method integrates physiological-state changes, behavioral performance, individual baseline deviation, and cross-modal coupling relationships to support the recognition and warning of normal, fatigue, stress, and abnormal-operation states.

### 1.2. Human-Factor Risk in Unmanned Intelligent System Operation

Although unmanned intelligent systems possess autonomous perception, decision-making, and control capabilities, human operators remain responsible for task setting, process monitoring, abnormality judgment, emergency response, and safety takeover [2,3,4,5]. In port supervision, inspection, and remote-operation scenarios, operators may need to manage dynamic task conditions, multi-device coordination, environmental disturbances, and sudden alarms. Under these circumstances, fatigue, stress, distraction, excessive workload, or reduced situational awareness may lead to delayed responses, judgment errors, unstable operation, or abnormal behaviors, thereby affecting operational safety and task-completion quality [4,5,6,7,11].

Operator human-factor risks are typically dynamic, concealed, and cumulative. Fatigue may result from prolonged monitoring, repetitive operation, low-stimulation supervisory tasks, or accumulated cognitive workload, and may be associated with reduced attention, slower responses, decreased operational stability, and increased errors [7,8,9,10,11,12]. Stress may arise during alarm handling, complex task switching, or emergency decision-making and may be reflected in changes in heart rate, heart-rate variability, electrodermal activity, respiration, and behavioral responses [13,14,21,22]. Abnormal-operation risk may further occur when the operator experiences excessive workload, misjudgment, nervousness, or unstable task conditions, and may be manifested by incorrect commands, trajectory deviation, task interruption, or untimely intervention.

Existing operator-state recognition methods mainly rely on physiological or behavioral information. Physiological signals, including heart rate, respiration, blood oxygen, electrodermal activity, electroencephalography, and eye-related measures, can reveal internal changes associated with fatigue, stress, and cognitive workload [7,8,9,10,11,12,13,14]. Behavioral features, such as control frequency, response delay, operation trajectory, command interval, erroneous operations, and task-completion time, directly reflect the operator’s external task-execution performance [11,16,17,18,19,20]. However, physiological signals may be affected by individual differences, sensor noise, motion artifacts, and environmental conditions [9,10,13,14], whereas behavioral abnormalities may appear only after the risk state has progressed to a certain level [11,16,17].

Therefore, single-modal methods have difficulty meeting the requirements for accurate, timely, and stable human-factor risk recognition in complex unmanned intelligent system operation scenarios. Multimodal sensing and information fusion can exploit the complementarity of physiological and behavioral data and improve the completeness and robustness of operator-state representation [16,17,18,19,20,23]. Behavioral telemetry has also been incorporated into real-time error-prediction and intervention frameworks, indicating that temporal, sequential, trajectory, and anomaly features can support proactive interaction-risk detection [24]. Nevertheless, existing physiological–behavioral fusion methods often rely on direct feature concatenation or simple decision-level fusion, and their capability to characterize physiological–behavioral coupling, multi-state risk evolution, model interpretability, and cross-operator generalization remains limited [18,19,20,21,22,23,24,25]. These limitations motivate the development of a more adaptive physiological–behavioral fusion method for dynamic human-factor risk assessment.

### 1.3. Bio-Inspired Physiological–Behavioral Fusion

When individuals face sustained task load, sudden events, or complex environmental stimuli, their physiological states and behavioral performance change jointly through stress response and feedback-regulation processes [13,14,21,22,26]. Fatigue may be associated with changes in heart-rate variability, respiratory rhythm, attention level, and response speed [7,8,9,10,11,12], whereas stress may be reflected in variations in heart rate, electrodermal activity, respiration, and behavioral responses [13,14,21,22]. Under higher-risk conditions, these internal-state changes may be accompanied by trajectory deviation, increased erroneous operations, delayed responses, or task interruption.

For operators of unmanned intelligent systems, task pressure, communication disturbances, continuous monitoring, multi-device coordination, and abnormal-event handling may jointly affect physiological load, cognitive state, and operational performance [4,5,27]. Physiological information can reveal internal-state variation, whereas behavioral information reflects the external execution of operational tasks. Their temporal association provides an important basis for assessing the formation and evolution of human-factor risks.

Accordingly, this study regards operator risk as a dynamic process involving physiological-state deviation, behavioral-performance deviation, and the coupling relationship between them. The proposed bio-inspired framework abstracts this process into a closed loop of “task stimulus–physiological response–behavioral performance–risk judgment–warning feedback.” It emphasizes individual baseline deviation because the same absolute physiological or behavioral value may have different implications for different operators [13,14,22,23]. It also emphasizes cross-modal coupling because fatigue, stress, and abnormal operation are often associated with concurrent changes in internal physiological states and external task performance [11,16,17,18,19,20].

Selective attention provides a further conceptual basis for adaptive fusion. Under different risk states, the relative importance of physiological and behavioral features may vary: fatigue may be more closely related to heart-rate variability, respiratory rhythm, and response delay; stress may be more strongly associated with heart rate, electrodermal activity, and manipulation frequency; and abnormal operation may depend more on trajectory deviation, erroneous operations, and task interruption [7,8,9,10,11,12,13,14,15,16,17,18,19,20,21,22]. Therefore, attention-based temporal modeling can be used to adaptively emphasize discriminative features and capture the temporal evolution of operator risk states. Recent advances in multimodal learning, attention mechanisms, and temporal models have demonstrated their value in fatigue detection, physiological-signal analysis, human–machine interaction monitoring, and real-time operator-state assessment [18,19,20,25,27,28,29]. On this basis, the proposed method integrates physiological–behavioral representation, cross-modal coupling, adaptive weighting, and temporal risk classification to support interpretable human-factor risk assessment in port-security scenarios.

Recent studies have provided further evidence for the value of multimodal operator-state assessment in safety-critical human–machine interaction. Huang et al. combined subjective ratings, physiological measures, and behavioral performance to assess mental workload under different task demands, demonstrating that internal-state and external-performance indicators can provide complementary evidence for workload assessment [30]. Walocha et al. employed eye-tracking, skin-conductance, and cardiovascular indicators to assess mental workload during automated-vehicle remote assistance, highlighting the importance of time-resolved multimodal monitoring in remote supervisory operations [31]. In collaborative robotics, Borghi et al. developed a multimodal approach for operator-stress assessment and showed that the integration of multiple information sources can support operator-centered safety evaluation [32]. Luzzani et al. further investigated synchronized ECG, respiration, fNIRS, and eye-tracking signals for stress and mental-workload monitoring in human–machine interaction, emphasizing the importance of cross-modal synchronization and temporal information fusion [33].

Comparable research in aerospace scenarios has also begun to investigate cross-modal physiological fusion for cognitive-workload assessment. Wang et al. proposed a cross-modal transformer framework for multimodal physiological signal fusion in aerospace training, demonstrating the relevance of dynamic cross-modal interaction modeling to safety-critical operator-state assessment [34]. Alreshidi et al. systematically reviewed the use of machine learning and psychophysiological data in aviation safety and identified workload, fatigue, stress, data preprocessing, and model interpretability as key issues for operator-centered safety applications [35]. These findings are directly relevant to the present study because imported unmanned intelligent system operators similarly perform safety-critical monitoring, decision-making, and intervention tasks under dynamic workload conditions.

Recent reviews have also emphasized the importance of physiological sensing and wearable technologies for workload assessment in human–machine interaction. Luzzani et al. reviewed physiological measures for mental-workload assessment in aviation and highlighted the complementary roles of cardiovascular, respiratory, neurophysiological, and ocular indicators [36]. Iarlori et al. summarized wearable-sensor-based approaches for cognitive-workload estimation in human–machine interaction and identified multimodal integration, individual variability, and real-time applicability as important research directions [37]. Collectively, these studies support the proposed bio-inspired physiological–behavioral fusion framework. In particular, they justify the joint use of complementary physiological and behavioral information, individual baseline consideration, adaptive feature weighting, cross-modal coupling, and temporal modeling for interpretable human-factor risk assessment in port-security scenarios.

### 1.4. Contributions of This Study

The main contributions of this study are as follows:

(1) A bio-inspired human-factor risk assessment framework is proposed for imported unmanned intelligent system operators in port-security scenarios, integrating physiological responses, behavioral performance, risk judgment, and warning feedback.
(2) A physiological–behavioral fusion model is developed to capture individual baseline deviations and cross-modal coupling relationships, while an attention mechanism is used to adaptively emphasize discriminative features under different risk states.
(3) A temporal multi-state risk classification and interpretation approach is established for recognizing normal, fatigue, stress, and abnormal-operation states, thereby supporting differentiated warning and safety-intervention decisions.

## 2. Bio-Inspired Mechanism and Problem Formulation

### 2.1. Stress Response and Neural Feedback Mechanism

When the human body faces high-load tasks, sudden stimuli, and potential risks, it usually experiences a dynamic process jointly composed of external stimulus perception, physiological-state changes, behavioral–response adjustment, and feedback regulation. This process reflects a typical stress-response and neural-feedback mechanism: external task pressure first acts on the human perceptual system and autonomic nervous system, causing changes in physiological indicators; subsequently, these internal-state changes further affect the individual’s response speed, movement stability, and operating behavior; finally, the nervous system realizes attention redistribution and behavioral correction through feedback regulation. For operators of imported unmanned intelligent systems at ports, continuous monitoring, remote control, abnormal-alarm handling, multi-device collaboration, and complex environmental changes may all induce similar physiological–behavioral responses, thereby forming human-factor risks such as fatigue, stress, delayed response, or abnormal operation.

From a bio-inspired perspective, operator human-factor risk is not the result of an abnormality in a single physiological indicator or a single operating behavior, but is jointly determined by physiological-state deviation, behavioral-performance deviation, and the coupling relationship between the two. Therefore, this manuscript divides the operator state into two aspects: physiological response and behavioral response. Let the physiological-state vector at time t be

(1) $$P_{t}=\left[HR_{t},RR_{t},S_{p}O2_{t},EDA_{t},HRV_{t}\right]$$

where $HR_{t}$, $RR_{t}$, $S_{p}O2_{t}$, $EDA_{t}$ and $HRV_{t}$ denote heart rate, respiratory rate, blood oxygen saturation, electrodermal activity, and heart rate variability, respectively. Let the behavioral-state vector at time t be

(2) $$B_{t}=\left[MF_{t},RD_{t},OT_{t},EC_{t},TCT_{t}\right]$$

where $MF_{t}$ $RD_{t}$ OT $EC_{t}$ and $TCT_{t}$ denote manipulation frequency, response delay, degree of operation-trajectory deviation, number of erroneous operations, and task-completion time, respectively.

To characterize the process in which physiological changes and behavioral abnormalities jointly induce human-factor risk, this manuscript defines the physiological–behavioral coupled risk-activation intensity as

(3) $$\Lambda_{t}=\sigma \left(\lambda_{p}{\left\|P_{t}-P^{0}\right\|}_{2}+\lambda_{b}{\left\|B_{t}-B^{0}\right\|}_{2}+\lambda_{pb}\Phi (P_{t},B_{t})\right)$$

where $\Lambda_{t}$ denotes the risk-activation intensity at time t; $P^{0}$ and $B^{0}$ denote the physiological baseline and behavioral baseline of the operator under the normal state, respectively; ${\left\|P_{t}-P^{0}\right\|}_{2}$ denotes the degree of deviation of the physiological state from the normal baseline; ${\left\|B_{t}-B^{0}\right\|}_{2}$ denotes the degree of deviation of the behavioral state from the normal baseline; $\Phi (P_{t},B_{t})$ denotes the coupling relationship between physiological changes and behavioral abnormalities; $\lambda_{p}$, $\lambda_{b}$, and $\lambda_{pb}$ are the weighting coefficients of physiological deviation, behavioral deviation, and the coupling term, respectively; and $\sigma \left(\cdot \right)$ is a normalized activation function.

To clarify the role of the following formulation, Equations (3)–(7) provide a conceptual bio-inspired representation of human-factor risk evolution rather than an independently implemented recursive module. The formulation illustrates how cumulative physiological deviation, behavioral-performance degradation, and prior risk conditions may jointly influence the current risk state. Its variables and thresholds are not separately learned or optimized in the subsequent neural-network experiments.

The purpose of this equation is to highlight the core assumption of this manuscript: operator human-factor risk is not directly determined by a single indicator, but is jointly activated by “physiological deviation–behavioral and deviation–physiological–behavioral coupling.” An increase in heart rate may only represent short-term nervousness, and a response delay may also be caused by increased task complexity. However, when abnormalities in physiological signals such as heart rate, respiration, and electrodermal activity occur simultaneously with behavioral abnormalities such as response delay, trajectory deviation, and increased erroneous operations, the probability that the operator enters a high-risk state increase significantly.

The physiological–behavioral coupling term can be expressed as

(4) $$\Phi (P_{t},B_{t})={\left(P_{t}-P^{0}\right)}^{T}W_{pb}\left(B_{t}-B^{0}\right)$$

where $W_{pb}$ is the coupling-weight matrix between physiological and behavioral features and is used to characterize the association strength between different physiological changes and different behavioral abnormalities. Unlike simple feature concatenation, this term is intended to describe how changes in internal physiological states are mapped to external operational risks. A decrease in heart rate variability may be associated with an increase in response delay, enhanced electrodermal activity may be associated with abnormal manipulation frequency, and disordered respiratory rhythm may be associated with fluctuations in the operation trajectory.

The human neural-feedback mechanism also indicates that risk states have temporal continuity and regulability. Fatigue risk usually has an accumulation effect; stress risk may be rapidly induced by sudden stimuli, and abnormal-operation risk may also be affected by the state at the previous moment and feedback intervention. Therefore, this manuscript further defines the dynamic update relationship of the risk state as

(5) $$R_{t}=\beta R_{t-1}+\left(1-\beta \right)\Lambda_{t}+\gamma F_{t-1}$$

where $R_{t}$ denotes the comprehensive human-factor risk level at time t; $R_{t-1}$ denotes the risk level at the previous moment; $\Lambda_{t}$ denotes the current risk-activation intensity; $F_{t-1}$ denotes the influence of warning or intervention feedback at the previous moment on the current state; β is the risk-memory coefficient; and γ is the feedback-regulation coefficient.

This formulation is used only to explain the theoretical risk-evolution mechanism. In the implemented method, temporal dependence is learned directly by the temporal classifier from the fused feature sequence; no closed-loop intervention signal is collected, modeled, or fed back during the reported experiments.

This equation is used to describe the dynamic evolution process of the operator’s risk state. Unlike static classification methods, this expression jointly incorporates historical risk, current risk activation, and feedback regulation into risk judgment, enabling the model to better characterize the accumulation of fatigue, the suddenness of stress, and the influence of warning feedback on subsequent states.

According to the comprehensive risk level $R_{t}$, the operator state can be further divided into different risk levels:

(6) $${\hat{Y}}_{t}=\left\{\begin{array}{l} 0,\;R_{t}<\theta_{1}\\ 1,\;\theta_{1}\leq R_{t}<\theta_{2}\\ 2,\;\theta_{2}\leq R_{t}<\theta_{3}\\ 3,\;R_{t}\geq \theta_{3} \end{array}\right.$$

where ${\hat{Y}}_{t}=0$ represents the normal state, ${\hat{Y}}_{t}=1$ represents fatigue risk, ${\hat{Y}}_{t}=2$ represents stress risk, and ${\hat{Y}}_{t}=3$ represents abnormal operation or a high-risk state; $\theta_{1}$, $\theta_{2}$, and $\theta_{3}$ are the thresholds for risk-level determination.

In summary, this manuscript abstracts the operator human-factor risk assessment process as

(7) $$\Phi (P_{t},B_{t})\to \Lambda_{t}\to R_{t}\to {\hat{Y}}_{t}$$

This process corresponds to “physiological-state changes and behavioral responses–risk activation–feedback regulation–risk-level judgment” in the human stress-response and neural-feedback mechanism. This mechanism provides a theoretical basis for the subsequent bio-inspired physiological–behavioral fusion model and also distinguishes the proposed method from simple physiological monitoring, behavioral recognition, or simple multimodal feature-concatenation methods.

It should be emphasized that the above stress-response and risk-evolution formulation provides a conceptual design rationale rather than a mathematically implemented biological model. The biological rationale motivates the use of individual baseline deviation, physiological–behavioral joint representation, and temporal risk evolution. In contrast, the feature encoders, bilinear coupling representation, attention mechanism, LSTM temporal model, and cross-entropy optimization used in the subsequent implementation are conventional machine-learning components. They are adopted as engineering tools and are not claimed to be direct mathematical derivations or faithful reproductions of biological mechanisms.

### 2.2. Bio-Inspired Mapping for Operator Risk Assessment

Based on the analysis of stress responses and neural-feedback mechanisms, operator human-factor risk assessment can be understood as a bio-inspired closed-loop process jointly composed of multi-source perception, state recognition, risk judgment, and feedback regulation. Under complex task environments, the human body perceives changes in internal states through physiological responses, reflects external response capabilities through behavioral performance, and regulates attention and behavioral strategies through neural-feedback mechanisms. Correspondingly, in imported unmanned intelligent system operation scenarios at ports, operator human-factor risk assessment should also simultaneously consider internal physiological states, external operating behaviors, task pressure, and risk–feedback results.

To establish the correspondence between biological mechanisms and engineering models, this manuscript maps human stress-response and neural-feedback processes into five functional modules in operator risk assessment: task-stimulus input, physiological-state perception, behavioral-response representation, physiological–behavioral fusion, and risk classification and feedback. The specific mapping relationships are shown in Table 1.

It should be emphasized that the proposed method is not intended to reproduce biological neural processes literally. Its bio-inspired nature lies in the functional mapping of biological principles to engineering design. First, baseline normalization reflects the homeostatic principle that an operator’s current state should be interpreted relative to an individual normal state rather than by using absolute physiological thresholds alone. Second, physiological–behavioral coupling reflects the coordinated relationship between internal stress responses and external operational performance. Third, adaptive feature weighting is inspired by selective attention, because the relative importance of physiological and behavioral information varies across fatigue, stress, and abnormal-operation states. Finally, the graded risk output and associated warning support represent an engineering interpretation of feedback regulation. Therefore, the attention mechanism itself is not claimed as the biological innovation; rather, it serves as an implementation mechanism for the proposed bio-inspired physiological–behavioral risk-assessment framework.

Table 1 presents a conceptual correspondence between biological processes and engineering functions; it does not imply a one-to-one biological implementation. Specifically, individual baseline deviation is motivated by the homeostatic nature of physiological regulation, and physiological–behavioral joint representation is motivated by the coexistence of internal-state changes and external behavioral responses under task pressure. The fusion operators, including the bilinear interaction representation and modality-level attention, are conventional engineering mechanisms used to model cross-modal association and adaptive weighting. Likewise, the LSTM is employed as a standard temporal classifier to capture sequential dependence, rather than as a model of biological neural dynamics. In practical modeling, the operator risk assessment process can be summarized into three levels.

The first level is the perception layer. This layer corresponds to the human process of perceiving external stimuli and internal-state changes. For unmanned intelligent system operators, the perception layer mainly includes two types of inputs. One type is physiological sensing data, such as heart rate, respiration, blood oxygen, electrodermal activity, and heart rate variability. The other type is behavioral sensing data, such as manipulation frequency, response delay, operation-trajectory deviation, number of erroneous operations, and task-completion time. Through this layer, basic state information of the operator under the current task environment can be obtained.

The second level is the fusion layer. This layer corresponds to the integration and selective-attention process of the human nervous system for multi-source information. The importance of physiological and behavioral features differs under different risk states. Fatigue is more likely to be manifested by decreased heart rate variability, changes in respiratory rhythm, and increased response delay. Stress is more likely to be manifested by increased heart rate, enhanced electrodermal activity, and abnormal manipulation frequency. Abnormal-operation states rely more on behavioral features such as trajectory deviation, number of erroneous operations, and interruption of task execution. Therefore, the fusion layer needs to adaptively weight different features rather than adopting fixed weights or simple concatenation.

The third level is the assessment layer. This layer corresponds to risk judgment and feedback regulation in human neural feedback. The model outputs operator-state categories and risk levels according to the fused risk representation and further provides a basis for warnings, interventions, or safety takeover. This layer focuses not only on the current risk state, but also on the temporal continuity and feedback-regulation effects of the risk state, thereby adapting to complex processes such as fatigue accumulation, sudden stress, and abnormal-operation evolution.

To describe the above mapping relationship more clearly, this manuscript formally represents the operator risk assessment process as

(8) $$X_{t}=M(P_{t},B_{t},C_{t})$$

where $X_{t}$ represents the comprehensive risk representation at time t, P represents the physiological-state vector, $B_{t}$ represents the behavioral-state vector, and $C_{t}$ represents task-context information such as task load, alarm state, and operational-scenario complexity. M(⋅) represents the bio-inspired mapping function used to map physiological responses, behavioral responses, and task context into a unified human-factor risk representation.

Furthermore, the risk assessment result can be expressed as

(9) $${\hat{Y}}_{t}=g(X_{t})$$

where ${\hat{Y}}_{t}$ represents the operator risk level and g(⋅) represents the risk-classification function. This expression indicates that the risk assessment in this manuscript is not directly performed according to a single physiological or behavioral indicator, but is based on the comprehensive representation of physiology–behavior–task context.

Based on the above bio-inspired mapping, the subsequent methodological section of this manuscript focuses on solving three problems. First, how to extract effective features capable of characterizing human-factor risks from physiological signals and behavioral data. Second, how to model physiological deviation, behavioral deviation, and the coupling relationship between them. Third, how to achieve adaptive selection of key features under different risk states through an attention mechanism. Through this bio-inspired mapping mechanism, operator human-factor risk assessment can shift from single-state recognition to multi-source fusion, dynamic perception, and interpretable classified judgment.

### 2.3. Problem Definition

The research object of this manuscript is operator human-factor risk assessment under imported unmanned intelligent system operation scenarios at ports. Unmanned intelligent systems include typical equipment such as unmanned aerial vehicles, unmanned ground vehicles, and unmanned surface vehicles. During task planning, remote control, status monitoring, abnormality handling, and safety takeover, operators’ physiological states and operating behaviors are affected by task load, environmental complexity, and sudden events, and may generate risk states such as fatigue, stress, delayed response, or abnormal operation. Therefore, the objective of this manuscript is to construct a bio-inspired fusion model capable of recognizing and classifying operator human-factor risks based on operator physiological signals and operating-behavior data.

Suppose that operator-state data are collected using fixed time windows during continuous operation. For the t-th time window, the operator physiological-state vector is expressed as $P_{t}=\left[p_{t,1},p_{t,2},\ldots ,p_{t,m}\right]$, where m denotes the number of physiological features, and typical features include heart rate, respiratory rate, blood oxygen saturation, electrodermal activity, and heart rate variability. The operator behavioral-state vector is expressed as $B_{t}=\left[b_{t,1},b_{t,2},\ldots ,b_{t,n}\right]$, where n denotes the number of behavioral features, and typical features include manipulation frequency, response delay, operation-trajectory deviation, number of erroneous operations, and task-completion time.

To simultaneously consider changes in the operator’s internal state and external operating performance, this manuscript jointly uses physiological features and behavioral features to form the multimodal input $X_{t}=\left[P_{t},B_{t},C_{t}\right]$, where $X_{t}$ represents the comprehensive operator-state feature at time t. If task-scenario information such as task load, abnormal alarms, operation duration, and environmental complexity is further considered, $C_{t}$ represents the task-context features. In practical modeling, whether this part of the information is introduced can be determined according to data availability.

Considering that operator states continuously change, a single time window may be insufficient to fully reflect the risk-formation process. Fatigue risk often gradually accumulates over time; stress risk may be rapidly triggered by sudden alarms, and abnormal-operation risk may also continuously evolve over a period of time. Therefore, this manuscript adopts T consecutive time windows to form the temporal input $X_{t-T+1:t}=\left[X_{t-T+1},X_{t-T+2},\ldots ,X_{t}\right]$. This sequence is used to describe the operator-state change process during a period before the current time and provides a basis for the subsequent model to learn risk-evolution patterns.

The objective of this manuscript is to learn a risk-assessment function f(⋅) to predict the operator’s human-factor risk category at the current moment according to the multimodal-state input within consecutive time windows:

(10) $${\hat{Y}}_{t}=f(X_{t-T+1:t})$$

During model training, given the training sample set $D={\left\{\left(X_{t-T+1:i},Y_{i}\right)\right\}}_{i=1}^{N}$, where N denotes the number of training samples, $X_{t-T+1:i}$ denotes the continuous multimodal-state sequence corresponding to the i-th sample, and Y denotes its true risk label; the model-training objective is to minimize the classification loss between the predicted results and the true labels:

(11) $$\underset{\Theta }{\min}\frac{1}{N}\sum_{i=1}^{N}L(Y_{i},{\hat{Y}}_{t})$$

where Θ denotes the model parameters and L(⋅) denotes the classification loss function, for which the cross-entropy loss is used. This optimization objective constrains the model to learn the discriminative patterns of different risk states from physiological–behavioral time-series data.

The problem defined in this manuscript is not a simple single-modal state-classification problem, but a multimodal temporal risk-assessment problem for complex operating scenarios. Its core lies in constructing a bio-inspired physiological–behavioral fusion model using operator physiological features, behavioral features, and optional task-context features during continuous operation, so as to realize dynamic recognition, risk classification, and warning support for normal, fatigue, stress, and abnormal-operation states.

In addition to classification accuracy, this manuscript also focuses on fusion effectiveness and risk interpretability of the model. Therefore, the problem definition includes not only “identifying which risk state the operator is in,” but also the following three aspects.

First, how to improve the recognition stability of fatigue, stress, and abnormal-operation states through joint modeling of physiological and behavioral features.

Second, how to characterize physiological-state deviation, behavioral-performance abnormalities, and the coupling relationship between them, so as to avoid insufficient information utilization caused by simple feature concatenation.

Third, how to identify key features under different risk states through an attention mechanism or weighting method so that the model output has a certain degree of interpretability.

In summary, the problem addressed in this manuscript can be summarized as follows: under imported unmanned intelligent system operation scenarios at ports, given the operator’s physiological signals, operating behaviors, and optional task-context data within consecutive time windows, a bio-inspired physiological–behavioral fusion model is learned to realize dynamic recognition, risk classification, and key-feature interpretation of the operator’s normal, fatigue, stress, and abnormal-operation states. This problem definition provides a basis for the subsequent model-structure design, experimental-scenario construction, and evaluation-metric selection.

## 3. Proposed Bio-Inspired Physiological–Behavioral Fusion Method

To address the problems of insufficient single-modal perception, inadequate characterization of physiological–behavioral coupling relationships, and difficulty in modeling the temporal evolution of risk states in human-factor risk recognition for imported unmanned intelligent system operators at ports, this manuscript proposes a bio-inspired physiological–behavioral fusion method. Inspired by human stress responses, neural feedback regulation, and multi-source perception mechanisms, the method jointly models changes in operator vital signs and operating-behavior performance. Through physiological-feature extraction, behavioral-feature extraction, coupled risk activation, temporal-state modeling, and risk-classification output, it realizes the recognition of normal, fatigue, stress, and abnormal-operation states.

Unlike traditional multimodal fusion methods, the proposed method does not simply concatenate physiological and behavioral features, but performs modeling at three levels. First, internal physiological-state features and external operating-behavior features of the operator are extracted separately. Second, physiological-state deviation, behavioral-state deviation, and the coupling relationship between them are characterized. Third, combined with state-evolution patterns within consecutive time windows, the operator’s risk state is dynamically recognized and classified. This method can better adapt to the characteristics of port unmanned intelligent system operation scenarios, such as rapid changes in task load, strong concealment of risk states, and obvious fluctuations in operator states.

The proposed method mainly consists of five modules: a data-input and preprocessing module, a physiological-feature extraction module, a behavioral-feature extraction module, a bio-inspired physiological–behavioral fusion module, and a human-factor risk-classification module. The overall process is shown in the following framework.

### 3.1. Overall Framework

The bio-inspired physiological–behavioral fusion method proposed in this manuscript is oriented toward human-factor risk-recognition tasks during the operation of imported unmanned intelligent systems at ports. Its overall design is guided by the conceptual sequence of ‘physiological perception–behavioral response–multi-source fusion–risk judgment–warning support’ under complex task environments. This sequence serves as an engineering design rationale and does not imply that the model reproduces a biological feedback mechanism. The method takes physiological signals, operating-behavior data, and optional task-context information during continuous operator operation as inputs and realizes the recognition of normal, fatigue, stress, and abnormal-operation states through dual-branch feature extraction, physiological–behavioral coupling representation, and risk-classification judgment.

**Figure 1:**
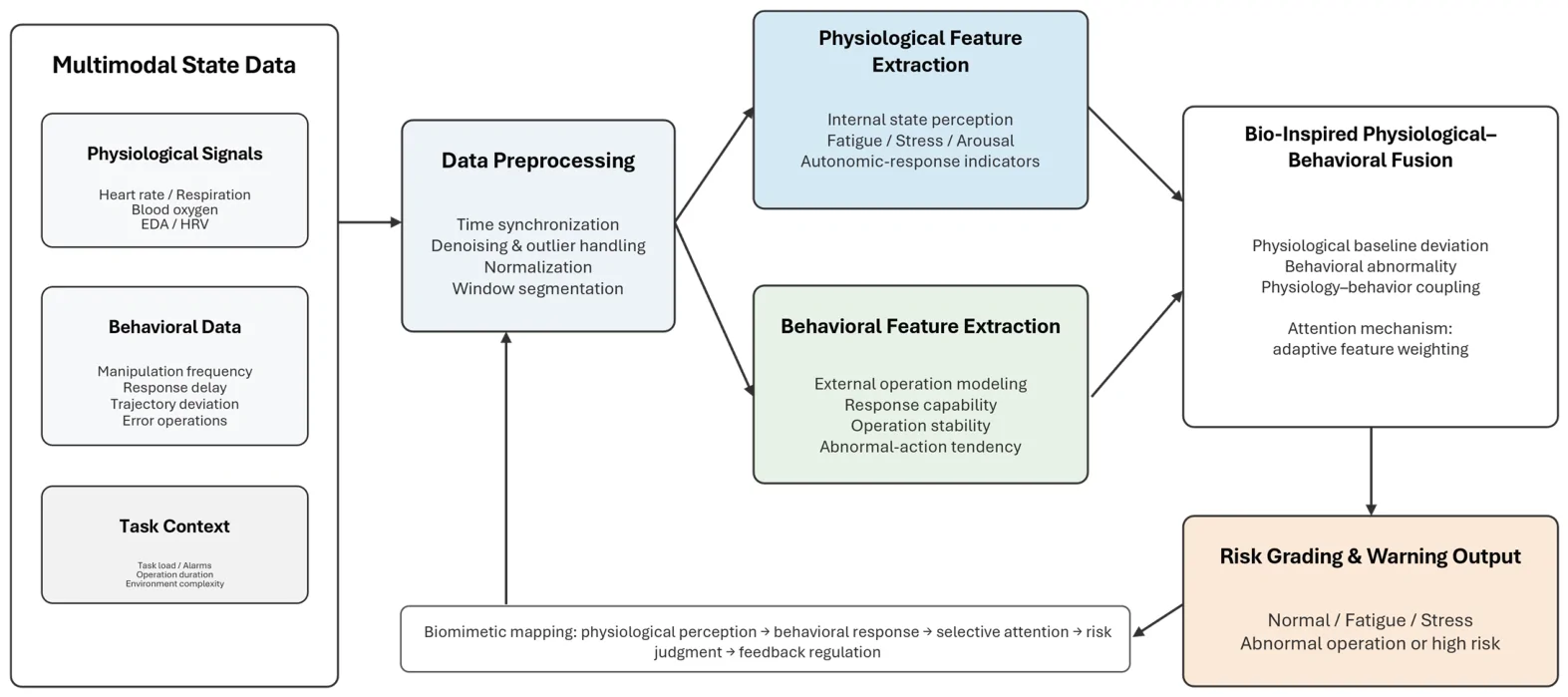
Bio-Inspired physiological–behavioral fusion framework for human-factor risk assessment.

The overall framework consists of five modules, as shown in Figure 1.

Figure 1 presents the overall architecture of the proposed bio-inspired physiological-behavioral fusion framework. To further clarify the operational sequence of the framework and the relationship among its main modules, Algorithm 1 summarizes the complete implementation procedure. The algorithm starts with synchronized physiological, behavioral, and task-event records, performs data-quality control and individual-baseline normalization, constructs temporal multimodal samples, and subsequently extracts physiological and behavioral representations.

The extracted representations are used to characterize physiological-state deviation, behavioral-performance deviation, and their coupling relationship. An attention-based fusion module then adaptively integrates the multimodal information, and a temporal-classification module predicts the current operator risk state. Finally, the predicted state is mapped to a graded warning action, including normal-status monitoring, fatigue warning, stress warning, or high-level safety-intervention support for abnormal operation. The following pseudocode provides an implementation-oriented summary of the proposed method; the detailed mathematical formulations of each module are presented in the subsequent sections.
**Algorithm 1:** Training procedure for the proposed bio-inspired physiological–behavioral fusion model.Input:  Physiological records P, behavioral records B, task-event records E;  individual normal-operation baseline R;  trained feature-extraction, fusion, and temporal-classification model M;  warning policy W. Output:  Risk state y ∈ {normal, fatigue, stress, abnormal operation};  warning action a. 1: Synchronize P, B, and E at the predefined sampling interval. 2: Remove incomplete, invalid, or unsynchronized records. 3: Normalize physiological and behavioral features relative to the individual baseline R. 4: Segment the synchronized records into consecutive temporal sequences. 5: Extract physiological features Xp and behavioral features Xb from each sequence. 6: Estimate physiological deviation, behavioral deviation, and their coupling representation Xc. 7: Apply attention-based adaptive weighting to obtain the fused representation Xf. 8: Use the temporal-classification module M to predict the current risk state y. 9: Generate a warning action a according to W:    if y = normal, output normal-status monitoring;    if y = fatigue, output low-level fatigue warning;    if y = stress, output medium-level stress warning;    if y = abnormal operation, output high-level warning and safety-intervention support. 10: Output y and a.

The following subsections introduce the specific implementation methods of physiological-feature extraction, behavioral-feature extraction, the bio-inspired fusion mechanism, and the human-factor risk-classification model.

### 3.2. Physiological Feature Extraction

The physiological-feature extraction module is used to characterize changes in the operator’s internal state during operation. In this manuscript, heart rate, respiratory rate, blood oxygen saturation, electrodermal activity, and heart rate variability are selected as physiological inputs to capture fatigue, stress, and changes in physiological load. Let a physiological signal within the t-th time window be

(12) $$S_{t}^{p}=\left\{s_{t,1}^{p},s_{t,2}^{p},\ldots ,s_{t,l}^{p}\right\}$$

where l denotes the number of sampling points within the window.

To reduce the effects of dimensional differences and individual differences, the signal is first standardized as

(13) $${\tilde{s}}_{t,i}^{p}=\frac{s_{t,i}^{p}-\mu_{p}}{\sigma_{p}}$$

where $\mu_{p}$ and $\sigma_{p}$ denote the mean and standard deviation of the corresponding physiological signal, respectively.

Within each time window, this manuscript mainly extracts three types of physiological features: physiological level, dynamic change, and baseline deviation. For any physiological indicator p, its features are expressed as

(14) $${\tilde{p}}_{t}=\frac{1}{l}\sum_{i=1}^{l}{\tilde{s}}_{t,i}^{p},\;\Delta p_{t}={\tilde{p}}_{t}-{\tilde{p}}_{t-1},\;d_{t}^{p}=\left|{\tilde{p}}_{t}-p^{0}\right|$$

where ${\tilde{p}}_{t}$ denotes the average physiological level of the current window, $\Delta p_{t}$ denotes the magnitude of change between adjacent windows, and $d_{t}^{p}$ denotes the degree of deviation of the current state from the normal physiological baseline $p^{0}$.

Finally, the physiological-feature vector of the t-th time window is expressed as

(15) $$F_{t}^{p}={\left[{\tilde{p}}_{t},\Delta p_{t},d_{t}^{p}\right]}_{p\in P}$$

where P is the set of physiological indicators. This feature vector is used to describe the level changes, trend changes, and individual-baseline deviations of the operator’s physiological state and provides input for subsequent physiological–behavioral fusion.

### 3.3. Behavioral Feature Extraction

The behavioral-feature extraction module is used to characterize the external behavioral responses of the operator during unmanned intelligent system operation. Unlike physiological features, which focus on reflecting internal states, behavioral features can directly characterize the operator’s response capability, operating stability, and tendency toward abnormal operation. In this manuscript, indicators such as manipulation frequency, response delay, operation-trajectory deviation, number of erroneous operations, and task-completion time are selected as the main behavioral inputs.

Let the operator behavioral data within the t-th time window be represented as

(16) $$S_{t}^{b}=\left\{s_{t,1}^{b},s_{t,2}^{b},\ldots ,s_{t,l}^{b}\right\}$$

where l denotes the number of behavioral sampling points or operation records within the window, and $s_{t,l}^{b}$ denotes the i-th behavioral observation. To eliminate differences in dimensions and value ranges among different types of behavioral indicators, the behavioral data are standardized as

(17) $${\tilde{s}}_{t,i}^{b}=\frac{s_{t,i}^{b}-\mu_{b}}{\sigma_{b}}$$

where $\mu_{b}$ and $\sigma_{b}$ denote the mean and standard deviation of the corresponding behavioral indicator, respectively.

Within each time window, this manuscript mainly extracts three types of behavioral features: behavioral level, behavioral change, and behavioral-baseline deviation. For any behavioral indicator b, its features are expressed as

(18) $${\tilde{b}}_{t}=\frac{1}{l}\sum_{i=1}^{l}{\tilde{s}}_{t,i}^{b},\;\Delta b_{t}={\tilde{b}}_{t}-{\tilde{b}}_{t-1},\;d_{t}^{b}=\left|{\tilde{b}}_{t}-b^{0}\right|$$

where ${\tilde{b}}_{t}$ denotes the average behavioral level within the current window, $\Delta b_{t}$ denotes the magnitude of behavioral change between adjacent windows, and $d_{t}^{b}$ denotes the degree of deviation of the current behavioral state from the normal behavioral baseline $b^{0}$.

Finally, the behavioral-feature vector of the t-th time window is expressed as

(19) $$F_{t}^{b}={\left[{\tilde{b}}_{t},\Delta b_{t},d_{t}^{b}\right]}_{b\in B}$$

where B denotes the set of behavioral indicators, including manipulation frequency, response delay, operation-trajectory deviation, number of erroneous operations, and task-completion time. This feature vector reflects the current level of the operator’s operating behavior, short-term change trends, and the degree of deviation from the normal operating baseline, and provides behavioral-side input for subsequent physiological–behavioral fusion modeling.

### 3.4. Attention-Based Multimodal Fusion

Based on the aforementioned physiological and behavioral features, this manuscript further constructs a multimodal fusion module based on an attention mechanism. The core objective of this module is to adaptively adjust the weights of physiological and behavioral features according to differences in the importance of various features under different risk states, thereby avoiding interference from redundant information caused by simple feature concatenation.

Let the physiological and behavioral features extracted from the t-th time window be $F_{t}^{p}$ and $F_{t}^{b}$, respectively. First, the two are mapped into a unified feature space:

(20) $$H_{t}^{p}=\phi_{p}(F_{t}^{p}),H_{t}^{b}=\phi_{b}(F_{t}^{b})$$

where $\phi_{p}(\cdot )$ and $\phi_{b}(\cdot )$ denote the feature-mapping functions of the physiological branch and behavioral branch, respectively, and $H_{t}^{p}$ and $H_{t}^{b}$ denote the mapped physiological-feature representation and behavioral-feature representation, respectively.

To characterize the contribution of different modalities to the current risk state, this manuscript introduces attention weights

(21) $$\alpha_{t}^{p},\alpha_{t}^{b}=Soft\max(\left[w^{T}H_{t}^{p},w^{T}H_{t}^{b}\right])$$

where $\alpha_{t}^{p}$ and $\alpha_{t}^{b}$ denote the weights of the physiological modality and behavioral modality within the current time window, respectively, and $w^{pT}$ and $w^{bT}$ are learnable parameters. This mechanism can dynamically adjust the importance of different modalities according to the current state. Under fatigue, physiological changes and response delay may be more critical. Under abnormal-operation states, behavioral features such as trajectory deviation and number of erroneous operations may have higher weights.

The attention mechanism operates at the modality level. It adaptively assigns weights to the physiological representation and the behavioral representation before fusion, thereby reflecting their relative contributions to the final risk-state prediction. These modality-level attention coefficients should not be interpreted as importance weights for individual input features.

The fused multimodal risk feature is expressed as

(22) $$F_{t}^{m}=\alpha_{t}^{p}H_{t}^{p}+\alpha_{t}^{b}H_{t}^{b}$$

where $F_{t}^{m}$ denotes the fused feature of the t-th time window. This representation contains both the operator’s internal physiological state and external behavioral performance and highlights key modalities that are more relevant to the current risk state through the attention mechanism.

To adapt to continuous changes in operator state, this manuscript inputs the fused features within consecutive time windows into the subsequent risk-classification model:

(23) $$F_{t-T+1:t}^{m}=\left[F_{t-T+1}^{m},F_{t-T+2}^{m},\ldots ,F_{t}^{m}\right]$$

This sequential feature can be used to characterize fatigue accumulation, sudden stress, and abnormal-operation evolution, thereby providing a more stable multimodal representation for subsequent human-factor risk-state recognition.

In summary, the attention-based multimodal fusion module realizes the transformation from physiological and behavioral features to a unified risk representation. Compared with fixed-weight fusion or simple concatenation, this method can dynamically adjust modal contributions according to different operating states and improve the model’s recognition capability and interpretability for complex human-factor risk states.

### 3.5. Biomimetic Feature Validation

The human-factor risk-classification module is used to further transform the multimodal features obtained through the aforementioned attention-based fusion into recognizable, interpretable, and applicable risk-level outputs. Since operator fatigue, stress, and abnormal-operation states usually have obvious temporal continuity, features within a single time window are often insufficient to completely reflect the risk-evolution process. Fatigue usually gradually accumulates during long-duration continuous operation; stress may be rapidly triggered by sudden alarms or complex tasks, whereas abnormal-operation states may continuously manifest over a period of time as response delay, trajectory deviation, and increased erroneous operations. Therefore, temporal modeling is introduced at the classification stage to comprehensively judge the fused features within consecutive time windows.

After attention-based fusion, the model obtains a multimodal risk representation within each time window. To characterize dynamic changes in operator state, this manuscript uses the fused features of T consecutive time windows as classification input. This input contains both the physiological–behavioral state at the current moment and the state-change trend during a preceding period, thereby helping the model identify the accumulation, suddenness, and persistence of risk states.

During human-factor risk classification, a temporal-modeling module is first used to extract state-evolution information from the consecutive fused features. Depending on the experimental conditions, this module can adopt structures such as LSTM, GRU, Transformer Encoder, or a one-dimensional convolutional neural network. Its purpose is not to re-extract single-modal features, but to learn the temporal variation patterns of different risk states from fused physiological–behavioral representations.

This process can be summarized as

(24) $$H_{t}=\varphi (F_{t-T+1:t}^{m})$$

where $F_{t-T+1:t}^{m}$ denotes the fused-feature sequence of T consecutive time windows, φ(⋅) denotes the temporal-modeling function, and $H_{t}$ denotes the high-level risk-state representation at the current moment. This representation integrates recent physiological changes, behavioral changes, and multimodal fusion information of the operator and serves as the direct input for subsequent risk classification.

At the risk-output stage, the high-level risk-state representation is input into the classification layer to obtain the predicted probabilities of different risk categories. The model finally selects the category with the highest probability as the human-factor risk state of the operator at the current moment.

This manuscript divides operator states into four categories: normal state, fatigue risk, stress risk, and abnormal operation or high-risk state. The normal state indicates that the operator’s physiological indicators and operating behaviors are within relatively stable ranges. Fatigue risk mainly corresponds to decreased attention, delayed response, and reduced operating stability after long-duration continuous operation. Stress risk mainly corresponds to nervous responses under sudden alarms, complex task switching, or high-load operations. Abnormal operation or a high-risk state corresponds to situations involving increased erroneous operations, obvious trajectory deviation, untimely response, or simultaneous abnormalities in multiple risk features.

During model training, a cross-entropy loss functions to constrain the consistency between predicted categories and true labels. Through training, the model can learn the mapping relationship between physiological–behavioral fusion features and risk categories under different risk states, thereby realizing automatic recognition of operator states. Compared with fixed-threshold-based discrimination methods, the data-driven classification model adopted in this manuscript can better adapt to human-factor risk differences among different operators, different task intensities, and different port-operation scenarios.

Finally, the output of the human-factor risk-classification module is used not only to determine the current operator state, but also as a basis for risk warning and safety intervention. When the model determines that the operator is in a fatigue or stress state, the system can prompt the operator to rest, reduce the task load, or increase operation confirmation. When the model determines that the operator is in an abnormal-operation or high-risk state, a higher-level warning, task suspension, manual review, or safety takeover can be triggered. In this way, the proposed method can transform physiological–behavioral fusion-recognition results into risk-classification and warning outputs for practical operating scenarios.

## 4. Experimental Design

To evaluate the proposed bio-inspired physiological-behavioral fusion method, experiments were conducted using synchronized physiological and operational-behavioral data collected during representative unmanned intelligent system operation tasks. The experimental protocol included scenario construction, data acquisition, risk-state labeling, participant-independent dataset partitioning, baseline comparison, and repeated computational evaluation. The experiments evaluated overall recognition performance, class-specific recognition capability, multimodal fusion effectiveness, warning reliability, and computational stability.

The underlying physiological and operational records are owned and managed by the relevant government authority and contain sensitive operational information. Therefore, individual-level physiological time series, detailed operation logs, fine-grained timestamps, and scenario-specific records are not publicly released. To ensure methodological transparency, this section reports the aggregate participant characteristics, sample distribution, sensing configurations, preprocessing procedures, data-partitioning strategy, model parameters, and repeated computational evaluation protocol.

### 4.1. Port-Based Operation Scenarios

To verify the recognition capability of the proposed method under different human-factor risk states, four types of experimental scenarios are constructed for typical port-operation tasks involving unmanned aerial vehicles, unmanned ground vehicles, and unmanned surface vehicles: normal operation, long-duration continuous operation, sudden abnormal-alarm handling, and complex multi-task collaboration. These scenarios are used to characterize the operator’s normal, fatigue, stress, and abnormal-operation or high-risk states, respectively.

In the normal-operation scenario, the operator completes equipment startup, path setting, status monitoring, and routine command operations according to a predefined procedure. No sudden alarms or obvious task conflicts are introduced during the experiment, so that the operator maintains relatively stable physiological and behavioral states. This scenario is mainly used to collect individual normal-state data and establish the physiological and behavioral baselines required for subsequent risk assessment.

The long-duration continuous-operation scenario is mainly used to simulate continuous monitoring and repetitive manipulation tasks. The operator is required to continuously perform target tracking, path correction, status confirmation, and information feedback over a relatively long period. As task duration increases, the operator may experience decreased attention, prolonged response time, slowed operation rhythm, and increased error rates. Therefore, this scenario is mainly used to obtain fatigue-risk samples and analyze the cumulative evolution characteristics of fatigue states.

The sudden abnormal-alarm handling scenario is used to simulate sudden events such as communication abnormalities, positioning deviations, obstacle alarms, trajectory abnormalities, or equipment faults. The operator is required to complete abnormality judgment, command input, and handling feedback within a limited time. This scenario induces stress responses by increasing task urgency and uncertainty and focuses on observing short-term changes in indicators such as heart rate, respiration, electrodermal activity, response delay, and manipulation frequency.

The complex multi-task collaboration scenario is used to simulate parallel monitoring of multiple devices, task switching, and priority adjustment during port operations. The operator is required to simultaneously process state information, trajectory corrections, and abnormal tasks of multiple unmanned systems and make decisions within a limited time. This scenario involves relatively high cognitive load and operational complexity and is prone to behaviors such as trajectory deviation, erroneous commands, task interruption, and delayed response. Therefore, it is mainly used to obtain samples of abnormal operation or high-risk states.

The correspondence between the four experimental scenarios and the target risk states is shown in Table 2.

Each point represents the metric value obtained from one computational run. Solid curves show run-specific metric values, horizontal dashed lines show the mean values across 250 runs, and shaded bands indicate the 95% confidence intervals of the mean. Statistical comparisons between the proposed method and baseline models were performed using paired two-sided Wilcoxon signed-rank tests with Holm–Bonferroni correction.

### 4.2. Data Collection and Labeling

**Figure 2:**
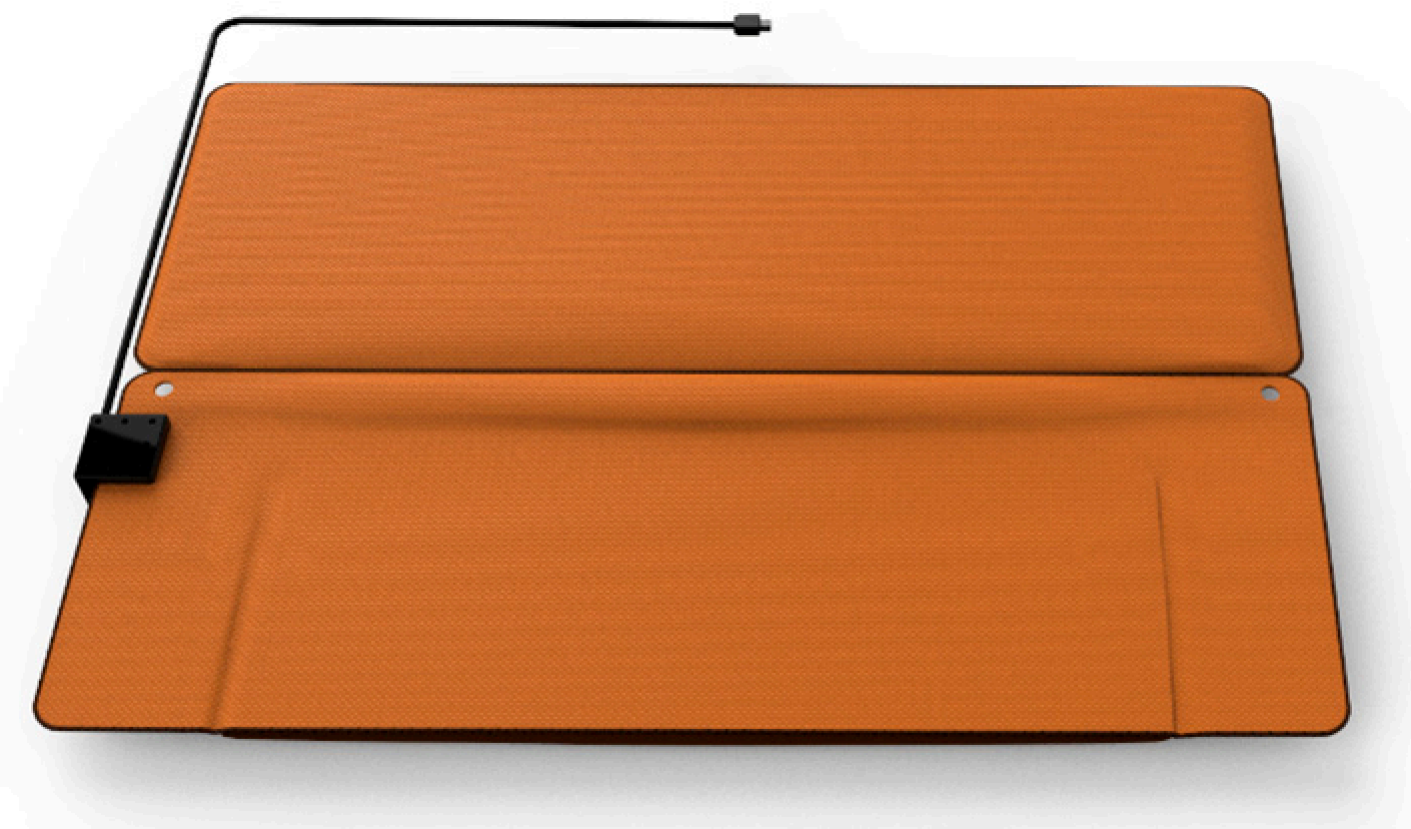
Non-invasive smart-cushion sensing system and sensor placement.

A non-invasive smart-cushion sensing system integrating MEMS and fiber-optic sensors was used to collect physiological data from the operators, thereby forming a multimodal sensing system for seated operation, as illustrated in Figure 2. The MEMS sensors were used primarily to acquire heart-rate and respiratory-rate signals, whereas the fiber-optic sensors captured dynamic pressure changes in the seat-contact area. The combined sensing configuration improved the stability and reliability of physiological monitoring under natural changes in seated posture.

The MEMS sensors were integrated into the backrest and seat-cushion regions to accommodate different postures during sustained console-based operation and to continuously acquire heart-rate- and respiratory-rate-related signals. Fiber-optic sensors were embedded in the pressure-contact area of the seat cushion to record dynamic changes in sitting posture and body-pressure distribution. This pressure information assisted in identifying posture-related signal disturbances and, together with operational-behavioral records and task-event information, provided supplementary evidence for human-factor risk analysis. Heart-rate variability was further derived from the heart-rate signal sequence. In addition, electrodermal activity was monitored using a wearable device positioned at the finger, wrist, or forearm to provide supplementary information on arousal and stress-related physiological changes.

To reduce signal deviations associated with individual body characteristics, posture variation, and environmental interference, an operator-specific normal-operation reference baseline was established from heart-rate and respiratory-rate records. The acquired data were calibrated relative to this baseline, and adaptive Kalman filtering was applied to suppress transient noise and motion-related disturbances. Sensor positions remained fixed during the experiment; the adaptive procedure adjusted signal-calibration and filtering parameters rather than the physical sensor placement. Records affected by sensor disconnection, incomplete signals, or evident motion artifacts were excluded during data-quality control.

Physiological and operational-behavioral data were collected from four trained operators during real port-operation tests involving representative unmanned intelligent system tasks. Data collection was conducted over approximately 10 operational days, with an effective recording duration of approximately 3 h per day. Therefore, the final dataset contained approximately 30 h of valid synchronized records. All physiological and behavioral records were sampled and synchronized at 10 s intervals.

Physiological data included heart rate, respiratory rate, blood oxygen saturation, electrodermal activity, and heart-rate variability. Behavioral data were obtained from the operation platform and task-event logs and included manipulation frequency, response delay, operation-trajectory deviation, erroneous operations, command corrections, and task-completion time. Response delay was defined as the time interval between the occurrence of a task instruction or alarm event and the operator’s first valid response.

Before analysis, the synchronized records were checked for data completeness and validity. Records affected by sensor disconnection, incomplete physiological signals, missing behavioral logs, or invalid task execution were excluded. The retained data were normalized relative to each operator’s normal-operation baseline before feature construction. After data-quality control, the final dataset contained 10,800 synchronized observations. The experimental schedule allocated approximately equals effective recording time to the four target risk states. Accordingly, the dataset included 2700 normal-state observations, 2700 fatigue-state observations, 2700 stress-state observations, and 2700 abnormal-operation observations. This approximately balanced sample distribution was used to reduce the potential influence of class imbalance on model training and evaluation.

Risk-state labels were determined through a multi-source procedure combining task-event records, operational conditions, safety-relevant task evidence, and independent expert review. No standardized self-report scale was administered during the real port-operation tests. Therefore, the labels of normal, fatigue, stress, and abnormal operation were not determined from a single questionnaire score; instead, they were assigned according to the temporal context of the task, recorded operational events, and independently reviewed safety-relevant evidence.

A normal-state candidate corresponded to stable task execution without abnormal alarms, safety events, or evident operational-performance degradation. A fatigue-state candidate was identified when long-duration continuous operation was accompanied by sustained reductions in response performance. A stress-state candidate was identified when sudden alarm handling or time-critical operational demands were accompanied by short-term deterioration in task-response performance. An abnormal-operation candidate was identified when the event records showed erroneous commands, marked trajectory deviation, task interruption, or delayed safety responses. Scenario identity was used as contextual information but was not treated as the sole label source.

The preliminary labels were independently reviewed by two experts, both of whom had more than 10 years of practical experience in unmanned-system operation and port-safety management. Each expert reviewed de-identified task-event records, operational conditions, and safety-relevant task summaries. The experts were blinded to the processed physiological feature values, model outputs, and model-prediction results during label assignment. Thus, the physiological and behavioral features used as model inputs were not used as the sole basis for determining the final labels.

Inter-rater agreement before consensus discussion was quantified using Cohen’s kappa. The agreement coefficient was κ = 0.76, indicating substantial agreement between the two experts. Disagreements were resolved through joint discussion. Samples for which a consensus label could not be reached were excluded from subsequent dataset construction, model training, and evaluation.

Because task scenarios were designed to elicit candidate risk states, task conditions remained partially associated with the risk labels in the present pilot dataset.

### 4.3. Baseline Methods

To comprehensively verify the effectiveness of the proposed method, comparison methods are selected from single-modal models, traditional machine-learning models, conventional multimodal fusion models, and temporal deep-learning models.

(1) Single-Modal Models

The physiological single-modal model uses only physiological features such as heart rate, respiration, blood oxygen, electrodermal activity, and heart rate variability for classification and is used to evaluate the capability of physiological information alone to recognize human-factor risks.

The behavioral single-modal model uses only behavioral features such as manipulation frequency, response delay, trajectory deviation, number of erroneous operations, and task-completion time for classification and is used to evaluate the discriminative capability of external operating behavior.

By comparing the proposed method with the two types of single-modal models, whether physiological–behavioral fusion can provide complementary information can be verified.

(2) Traditional Machine-Learning Models

Traditional classification models such as Support Vector Machine, Random Forest, and Extreme Gradient Boosting are selected as baselines. For these methods, physiological and behavioral features are first directly concatenated and then input into the classifier for risk-state recognition.

Traditional machine-learning models have characteristics such as simple structures and convenient interpretation, and can be used to compare the performance differences between deep temporal models and traditional static classification methods.

(3) Conventional Multimodal Fusion Models

Conventional multimodal fusion models include direct feature concatenation and fixed-weight fusion. Direct feature concatenation connects physiological and behavioral features and inputs them into a unified classifier, whereas fixed-weight fusion combines the two modal features according to predefined proportions.

Comparison with the above methods can verify the role of the attention mechanism in dynamically adjusting the contributions of physiological and behavioral modalities.

(4) Deep Temporal Models

To verify the temporal-modeling capability of the proposed model, LSTM, GRU, or Transformer Encoder can be selected as comparison models. These models use the same input features and data-partitioning method but do not introduce the bio-inspired coupling or modal-attention modules designed in this manuscript.

All comparison models should adopt consistent data preprocessing, training-set partitioning, and evaluation metrics to ensure comparability of the experimental results.

### 4.4. Evaluation Metrics

This manuscript evaluates model performance in terms of classification accuracy, category-recognition capability, false-alarm and missed-alarm conditions, and operational efficiency.

For the multi-class risk-recognition task, Accuracy is adopted to measure the proportion of overall predictions correctly made by the model. Since the number of samples in different risk categories may be imbalanced, Precision, Recall, and F1-score are further adopted to evaluate the model’s recognition capability for different risk categories. To prevent the normal category with a relatively large number of samples from dominating the overall results, the macro-average metric is also reported:

(25) $$\mathrm{Macro}\text{-}F1=\frac{1}{K}\sum_{k=1}^{K}F1_{k}$$

where K denotes the number of risk categories and $F1_{k}$ denotes the F1-score of the k-th category. Macro-F1 assigns equal weights to all categories and is therefore more suitable for evaluating the recognition capability for minority categories such as fatigue, stress, and abnormal operation.

In addition to conventional classification metrics, the False Alarm Rate (FAR) and Missed Detection Rate (MDR) were used to evaluate warning-related error characteristics. FAR represents the proportion of normal-state samples incorrectly classified as risk states, whereas MDR represents the proportion of risk-state samples that are not correctly identified. In port-security supervision, an excessive FAR may increase the workload associated with manual review and unnecessary intervention, whereas an excessive MDR may delay the identification of potentially unsafe operator states.

Table 3 summarizes the evaluation metrics used in this study. Because the present work focuses on offline risk-state classification, multimodal-fusion effectiveness, and repeated computational stability, the evaluation framework includes overall classification metrics, class-balanced metrics, and warning-reliability metrics. End-to-end online latency and deployment efficiency were not evaluated and are discussed as directions for future work.

In the subsequent result analysis, this manuscript further verifies the effectiveness and interpretability of the proposed method through overall performance comparison, recognition results under different risk states, ablation experiments, and attention-weight analysis.

For each model and evaluation metric, results are reported as the mean ± standard deviation across 250 repeated computational training-and-evaluation runs. The 95% confidence interval (CI) of the mean was calculated as the mean $\pm 1.96\times SD/\sqrt{250}$. To compare the proposed method with each baseline model, paired two-sided Wilcoxon signed-rank tests were performed on metric values obtained from runs using the same random-seed schedule. The resulting *p*-values were adjusted using the Holm–Bonferroni procedure to account for multiple comparisons. Statistical significance was defined as an adjusted *p*-value below 0.05.

### 4.5. Implementation Details and Baseline Settings

All baseline models and the proposed model were trained and evaluated using the same participant-level train/validation/test split, preprocessing procedure, feature inputs, and held-out test set. Hyperparameters were selected using the validation set only, and the test set was not used for model selection. For SVM, an RBF kernel was adopted with C = 10, γ = scale, and balanced class weights. The RF model used 300 trees, a maximum tree depth of 12, a minimum leaf size of 2, and the square-root rule for feature selection. XGBoost was configured with 300 estimators, a maximum depth of 5, a learning rate of 0.05, subsampling and column-sampling ratios of 0.8, and an $L_{2}$ regularization coefficient of 1.

The LSTM-fusion baseline used one LSTM layer with 128 hidden units and a dropout rate of 0.3. The proposed model used 64-dimensional physiological and behavioral representations, a modality-attention hidden dimension of 32, a 128-dimensional fused representation, and one LSTM layer with 128 hidden units and a dropout rate of 0.3. The neural models were trained using the Adam optimizer with a learning rate of 0.001, a batch size of 32, a maximum of 100 epochs, cross-entropy loss, and early stopping with a patience of 15 epochs based on validation performance. In the 250 repeated computational runs, the data split and hyperparameter configuration remained fixed. The predefined random-seed schedule controlled the stochastic initialization and mini-batch ordering of neural models and the corresponding stochastic procedures of applicable baseline models.

## 5. Results and Analysis

This section verifies the proposed bio-inspired physiological–behavioral fusion method in terms of overall recognition performance, recognition effects under different risk states, model ablation results, and feature interpretability. To ensure fair comparison, all models adopt the same data partitioning, preprocessing procedure, and training settings and are evaluated on an independent test set. The experimental results mainly examine the effects of physiological–behavioral fusion, the attention mechanism, and temporal modeling on the performance of operator human-factor risk recognition.

### 5.1. Overall Performance Comparison

To verify the overall effectiveness of the proposed bio-inspired physiological–behavioral fusion method, this manuscript compares it with traditional machine-learning models, single-modal recognition models, a direct feature-concatenation model, and a conventional temporal fusion model. All models adopt the same data partitioning, preprocessing procedure, and evaluation metrics to ensure fairness of the experimental results. The evaluation metrics include Accuracy, Precision, Recall, F1-score, Macro-F1, False Alarm Rate (FAR), and Missed Detection Rate (MDR). The overall performance comparison results are shown in Table 4.

Values are reported as mean ± standard deviation, with 95% confidence intervals of the mean shown in brackets. All models were evaluated using the same participant-independent data partition and the same 250 random-seed schedule. Adjusted *p*-values indicate paired two-sided Wilcoxon signed-rank tests between the proposed method and each baseline model, with Holm–Bonferroni correction for multiple comparisons.

The overall performance comparison results are shown in Table 4. All results in the table are expressed as the mean and standard deviation of 250 repeated computational training-and-evaluation runs, where the mean is used to reflect the average recognition performance of the model, and the standard deviation is used to measure the stability of the model across different trials. Higher Accuracy, Precision, Recall, F1-score, and Macro-F1 indicate better risk-recognition capability, whereas lower FAR and MDR indicate more reliable warning performance.

As shown in Table 4, the proposed method achieves the best results for all evaluation metrics. Its Accuracy, Precision, Recall, F1-score, and Macro-F1 reach 92.16% ± 0.47%, 92.43% ± 0.51%, 91.68% ± 0.54%, 92.04% ± 0.49%, and 90.86% ± 0.53%, respectively. Meanwhile, the FAR and MDR of the proposed method are 4.83% ± 0.34% and 6.74% ± 0.42%, respectively, both lower than those of the other comparison models. These results indicate that the proposed method not only has relatively high average classification performance, but also exhibits better stability and risk-warning reliability across repeated trials.

The overall performance of the traditional machine-learning models is relatively low. Among them, the Accuracy and Macro-F1 of SVM are 78.63% ± 1.25% and 76.94% ± 1.38%, respectively, and its standard deviations are relatively large among the models, indicating that the performance of the static classification method fluctuates considerably across different trials. The overall performance of Random Forest and XGBoost is improved, with XGBoost achieving an Accuracy of 84.76% ± 0.96%, but it is still lower than the multimodal temporal models. This indicates that although ensemble-learning methods can enhance the classification capability for nonlinear features, static inputs based only on a single time window still have difficulty fully capturing dynamic processes such as fatigue accumulation, stress triggering, and continuous evolution of abnormal operation.

Among the single-modal models, the Accuracy of the physiological-only model is 80.92% ± 1.18%, slightly higher than the 79.84% ± 1.22% of the behavioral-only model. Physiological signals can reflect internal-state changes such as operator fatigue, nervousness, and stress at an earlier stage, but they are susceptible to individual differences, sensor noise, and motion artifacts. Behavioral features can directly characterize external risk manifestations such as delayed response, trajectory deviation, and erroneous operations, but they usually have a certain lag. The Macro-F1 values of both single-modal models are below 80%, and their standard deviations are higher than those of the multimodal models, indicating that relying on a single information source makes it difficult to stably and comprehensively characterize operator human-factor risks.

The Accuracy and Macro-F1 of the direct feature-concatenation model reach 86.37% ± 0.82% and 84.96% ± 0.91%, respectively, which are significantly better than those of the two single-modal models, while their standard deviations are also reduced. This indicates that physiological and behavioral information have strong complementarity, and multimodal input can improve the completeness of risk-state representation and the stability of repeated trials. However, direct feature concatenation adopts a fixed fusion approach and cannot dynamically adjust the contributions of the physiological and behavioral modalities according to different risk states; therefore, its performance is still lower than that of the temporal fusion model.

The Accuracy of the LSTM-based fusion model reaches 89.56% ± 0.68%, which is 4.80 percentage points higher than that of XGBoost, while the standard deviation decreases from 0.96% to 0.68%. This result indicates that temporal modeling not only improves average recognition performance, but also reduces the influence of instantaneous fluctuations within a single time window on classification results, thereby more effectively characterizing state changes such as fatigue accumulation, sudden stress, and continuous evolution of abnormal operation.

On the basis of LSTM-based fusion, the proposed method further introduces physiological–behavioral coupling modeling and an attention mechanism, increasing Accuracy, F1-score, and Macro-F1 by 2.60, 2.70, and 2.69 percentage points, respectively. Meanwhile, their corresponding standard deviations decrease from 0.68%, 0.71%, and 0.76% to 0.47%, 0.49%, and 0.53%, respectively. This indicates that the physiological–behavioral coupling relationship and attention weighting not only enhance the risk-discrimination capability of the model, but also improve output stability under different repeated-trial conditions.

In terms of warning reliability, compared with the direct feature-concatenation model, the proposed method reduces FAR and MDR by 3.11 and 4.12 percentage points, respectively; compared with the LSTM-based fusion model, they are reduced by 1.71 and 2.22 percentage points, respectively. In addition, the standard deviations of FAR and MDR for the proposed method are only 0.34% and 0.42%, further indicating that the method can maintain relatively stable false-alarm and missed-detection control capabilities under different experimental conditions.

Overall, the results in Table 4 indicate that physiological–behavioral dual-modal input improves the completeness of operator-state representation, temporal modeling enhances the model’s capability to characterize the dynamic evolution of risks, and physiological–behavioral coupling and the attention mechanism further improve key-feature selection capability and model stability. Therefore, the proposed method outperforms the other comparison models in terms of average recognition performance, repeated-trial stability, and risk-warning reliability.

**Figure 3:**
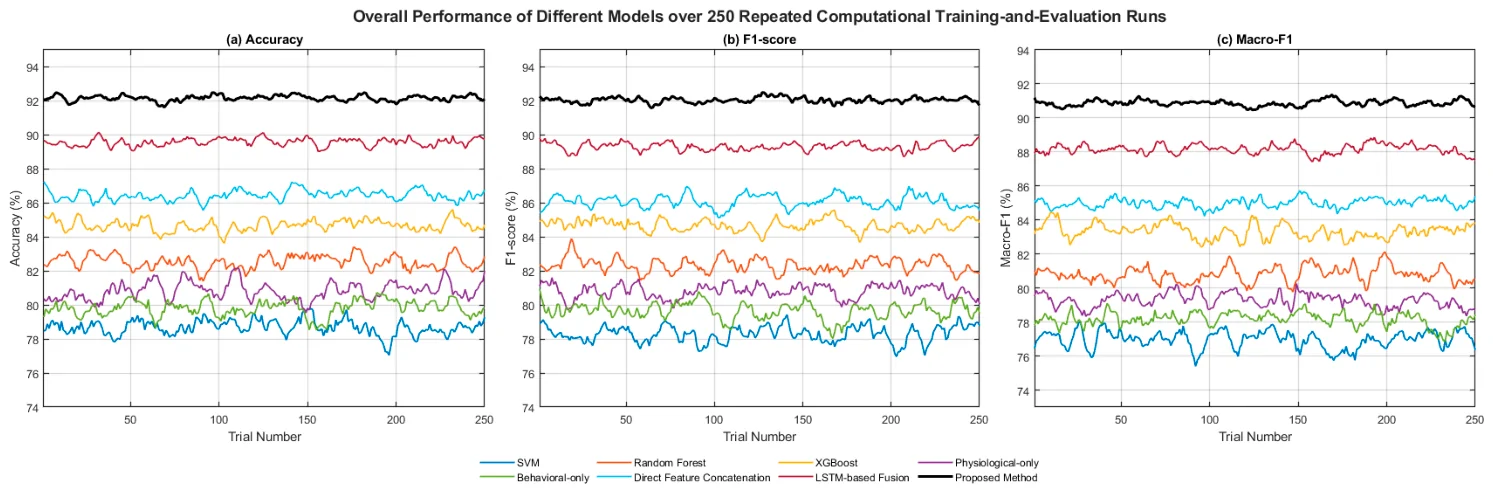
Overall performance curves of different models over 250 repeated computational training-and-evaluation runs.

To more intuitively compare the overall recognition performance of different models, this manuscript further visualizes the three evaluation metrics of Accuracy, F1-score, and Macro-F1. For each model, 250 repeated computational training-and-evaluation runs are conducted, and the corresponding metric results of each trial are recorded. Figure 3 takes the trial number as the horizontal axis and the evaluation metric value as the vertical axis, showing the performance variations and stability of different models during repeated trials. By comparing the overall levels and fluctuation amplitudes of the model curves, their recognition accuracy, class-balance capability, and result stability can be further analyzed.

As can be seen in Figure 3, there are relatively obvious differences in the overall performance of different models over the 250 repeated computational training-and-evaluation runs. The Accuracy, F1-score, and Macro-F1 curves of the traditional machine-learning models are generally at relatively low levels and have relatively large fluctuations. Among them, the average Accuracy and Macro-F1 of SVM are 78.63% and 76.94%, respectively. Although the three metrics of Random Forest and XGBoost are improved, they are still generally lower than those of the multimodal temporal models. This result indicates that relying only on static features within a single time window makes it difficult to fully characterize human-factor risk states with obvious temporal dependence, such as fatigue accumulation, stress triggering, and continuous evolution of abnormal operation.

The curves of the physiological-only and behavioral-only models are generally higher than those of SVM, but the performance improvement remains limited. The average Accuracy of the physiological-only model is 80.92%, slightly higher than the 79.84% of the behavioral-only model, and its F1-score and Macro-F1 show similar trends. Physiological signals can reflect internal-state changes such as operator fatigue, nervousness, and stress at an earlier stage, but they are easily affected by individual differences, sensor noise, and motion artifacts. Behavioral features can directly characterize external risk manifestations such as delayed response, trajectory deviation, and erroneous operations, but obvious changes usually occur only after the risk state has developed to a certain degree. Therefore, the performance curves of both single-modal models exhibit certain fluctuations, indicating that relying on a single information source makes it difficult to stably and completely describe operator human-factor risks.

The three metric curves of the direct feature-concatenation model are generally higher than those of the two single-modal models, with its average Accuracy and Macro-F1 reaching 86.37% and 84.96%, respectively, indicating strong complementarity between physiological and behavioral information. Meanwhile, the curve fluctuations of this model over the 250 repeated trials are also reduced, showing that multimodal input can improve the stability of risk recognition. However, direct feature concatenation assumes fixed importance for different modalities and features and cannot dynamically adjust information contributions according to operator states and task contexts. Therefore, its overall performance remains lower than that of the temporal fusion model and the proposed method.

The Accuracy, F1-score, and Macro-F1 curves of the LSTM-based fusion model are further improved, with significantly reduced overall fluctuations. Its average Accuracy reaches 89.56%, which is 4.80 percentage points higher than that of XGBoost. This indicates that continuous time-window modeling can more effectively capture the accumulation, suddenness, and persistence of operator states, thereby improving the model’s recognition capability for dynamic human-factor risks.

Among all comparison models, the three performance curves of the proposed method consistently remain at relatively high levels while showing smaller fluctuation amplitudes, demonstrating better recognition accuracy and repeated-trial stability. Its average Accuracy, F1-score, and Macro-F1 reach 92.16%, 92.04%, and 90.86%, respectively, which are 2.60, 2.70, and 2.69 percentage points higher than those of LSTM-based fusion. This result indicates that further introducing the physiological–behavioral coupling relationship and attention-weighting mechanism on the basis of temporal modeling can dynamically highlight physiological and behavioral information with higher discriminative value under different risk states, reduce interference from redundant features and noise, and thereby enhance the model’s recognition capability and result stability for complex human-factor risk states.

**Figure 4:**
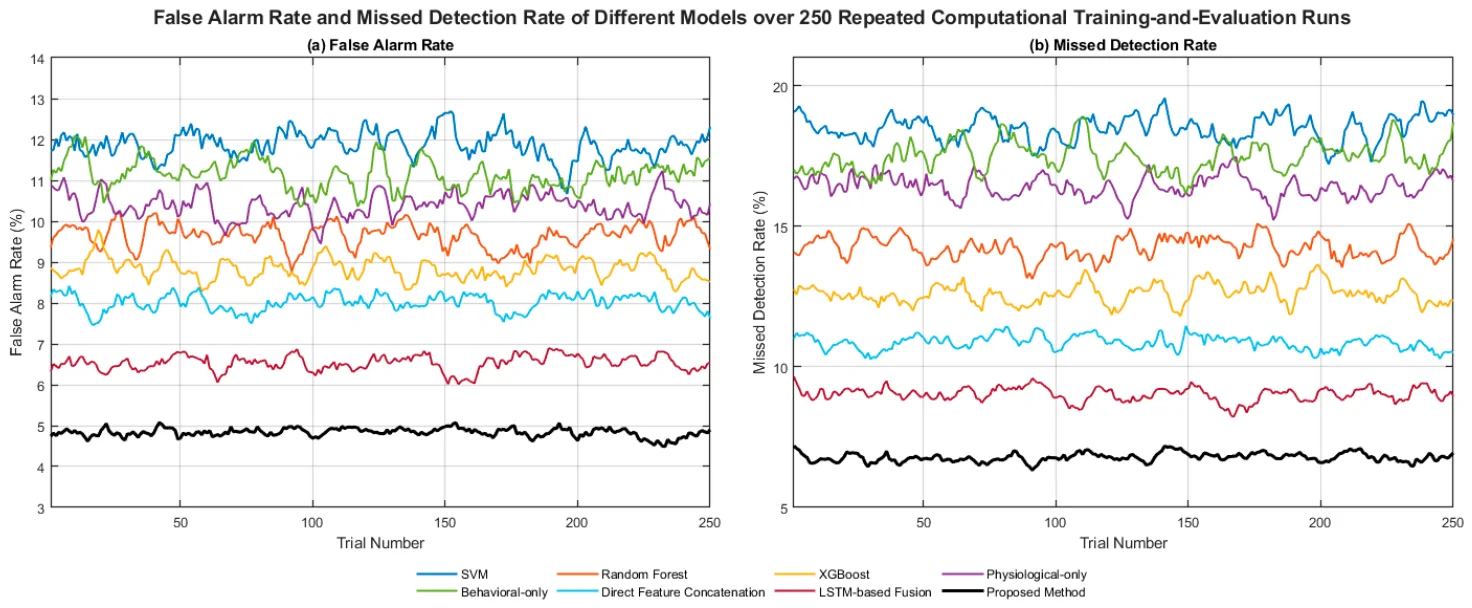
False alarm rate and missed detection rate curves of different models over 250 repeated computational training-and-evaluation runs.

In addition to overall classification performance, the False Alarm Rate (FAR) and Missed Detection Rate (MDR) are also important indicators for measuring the reliability of model risk warnings. FAR reflects the probability that the model incorrectly recognizes a normal state as a risk state, whereas MDR reflects the probability that the model fails to recognize a true risk state in a timely manner. A high false alarm rate increases the burden of manual review and safety handling, whereas a high missed detection rate may result in fatigue, stress, or abnormal-operation states not being detected in a timely manner. Therefore, this manuscript further compares FAR and MDR for different models over 250 repeated computational training-and-evaluation runs, as shown in Figure 4.

Each point represents the FAR or MDR obtained from one computational run. Solid curves show run-specific values, horizontal dashed lines show the mean values across 250 runs, and shaded bands indicate the 95% confidence intervals of the mean. Lower FAR and MDR indicate better warning reliability. Statistical comparisons between the proposed method and baseline models were performed using paired two-sided Wilcoxon signed-rank tests with Holm-Bonferroni correction.

As can be seen in Figure 4, there are obvious differences in the false alarm rates and missed detection rates of different models over the 250 repeated computational training-and-evaluation runs. The FAR and MDR curves of traditional machine-learning models and single-modal models are generally at relatively high levels and have relatively large fluctuations. Among them, the average FAR and MDR of SVM are 11.83% and 18.46%, respectively, while those of the behavioral-only model are 11.24% and 17.58%, respectively. This indicates that when a model relies only on static features or single-modal information, insufficient information representation can easily cause normal states to be incorrectly judged as risk states, while true risks such as fatigue, stress, and abnormal operation may also be missed.

Compared with the traditional models, the FAR and MDR of the direct feature-concatenation model decrease to 7.94% and 10.86%, respectively, and the overall fluctuations of its repeated-trial curves are reduced, indicating that joint input of physiological and behavioral information can improve risk-warning stability. However, the fixed concatenation approach has difficulty dynamically adjusting the contribution of each modality according to different risk states, and therefore certain false alarms and missed detections still exist.

The LSTM-fusion model reduced FAR and MDR to 6.54% and 8.96%, respectively, indicating that temporal modeling can capture state changes across consecutive time windows and reduce errors caused by instantaneous fluctuations. The proposed method achieved lower average FAR and MDR values of 4.83% and 6.74%, respectively, over 250 repeated computational runs, with smaller run-to-run variation.

Compared with direct feature concatenation, the proposed method reduced FAR and MDR by 3.11 and 4.12 percentage points, respectively; compared with LSTM fusion, the reductions were 1.71 and 2.22 percentage points. These results suggest that modality-level adaptive fusion of physiological and behavioral information improves warning-related performance and repeated-run stability. Overall, Figure 2 and Figure 3 show that the proposed method achieved higher classification performance and lower FAR and MDR under the evaluated experimental conditions.

### 5.2. Recognition Results Under Different Risk States

To further analyze the recognition capability of the proposed method for different types of human-factor risk states, this manuscript separately calculates the classification results for four states: normal, fatigue, stress, and abnormal operation. Compared with overall average metrics, class-specific results can more clearly reflect differences in model recognition under different risk states, and are particularly helpful for analyzing confusion between similar states such as fatigue and stress, as well as the model’s detection capability for high-risk states such as abnormal operation.

The classification results under different risk states are shown in Table 5. It should be noted that the results in the table are expressed as the mean ± standard deviation of 250 repeated computational training-and-evaluation runs.

Values are reported as mean ± standard deviation, with 95% confidence intervals of the mean shown in brackets. Precision, Recall, and F1-score were calculated separately for the normal, fatigue, stress, and abnormal-operation states. The reported variability reflects repeated stochastic model training and optimization under a fixed participant-independent test set.

As can be seen from Table 5, the proposed method achieves relatively high recognition performance for all four risk states. Among them, the Precision, Recall, and F1-score for the normal state reach 94.28%, 95.17%, and 94.72%, respectively, indicating that the model can relatively accurately distinguish the normal operating state from risk states. The relatively high Recall for the normal state also indicates that the model produces relatively few misclassifications of normal samples, which is consistent with the low false alarm rate reported in Section 5.1.

The F1-score for the abnormal-operation state reaches 92.81%, second only to that of the normal state, indicating that the proposed method can effectively identify obvious behavioral abnormalities such as trajectory deviation, increased erroneous commands, delayed response, and task interruption. When these behavioral abnormalities occur simultaneously with deviations in physiological states such as heart rate, respiration, and electrodermal activity, physiological–behavioral coupling modeling can form a stronger risk representation, thereby improving the discriminative capability for high-risk states.

In comparison, the recognition performance for fatigue and stress is slightly lower, with F1-scores of 90.59% and 89.97%, respectively. This is mainly because the two states have certain overlaps in some physiological and behavioral features. Both fatigue and stress may be accompanied by changes in heart rate variability, abnormal respiratory rhythm, increased response delay, and decreased operating stability. Therefore, the model may generate certain confusion during state-transition stages.

The Recall for fatigue is 89.84%, slightly higher than the 89.21% for stress. This indicates that the model recognizes persistent fatigue relatively stably. Fatigue is usually manifested by increased continuous operation time, gradual decreases in response speed, and accumulated operating errors. Its temporal characteristics are relatively obvious and are therefore easier to capture through continuous-window modeling. Stress, in contrast, is usually rapidly triggered by sudden alarms or complex task switching and has stronger instantaneous characteristics and individual differences, making its recognition relatively more difficult.

To further show the classification relationships among different risk states, this manuscript constructs the normalized confusion matrix of the proposed method, as shown in Table 6.

As can be seen from Table 6, confusion mainly occurs between fatigue and stress. Approximately 5.73% of fatigue samples are recognized as stress, while approximately 6.12% of stress samples are recognized as fatigue. This result is related to the similar manifestations of the two states in terms of heart-rate changes, respiratory rhythm, decreased attention, and response delay.

In comparison, the classification boundaries of normal and abnormal-operation states are relatively clear. The correct recognition rate for the normal state is 95.17%, with only a small number of samples incorrectly classified as fatigue or abnormal operation. The correct recognition rate for abnormal operation reaches 92.47%, and its main confusion category is stress. High-frequency correction, rapid operation, and trajectory fluctuations under certain stress states may exhibit characteristics similar to abnormal operation, thereby causing a small number of samples to be confused.

**Figure 5:**
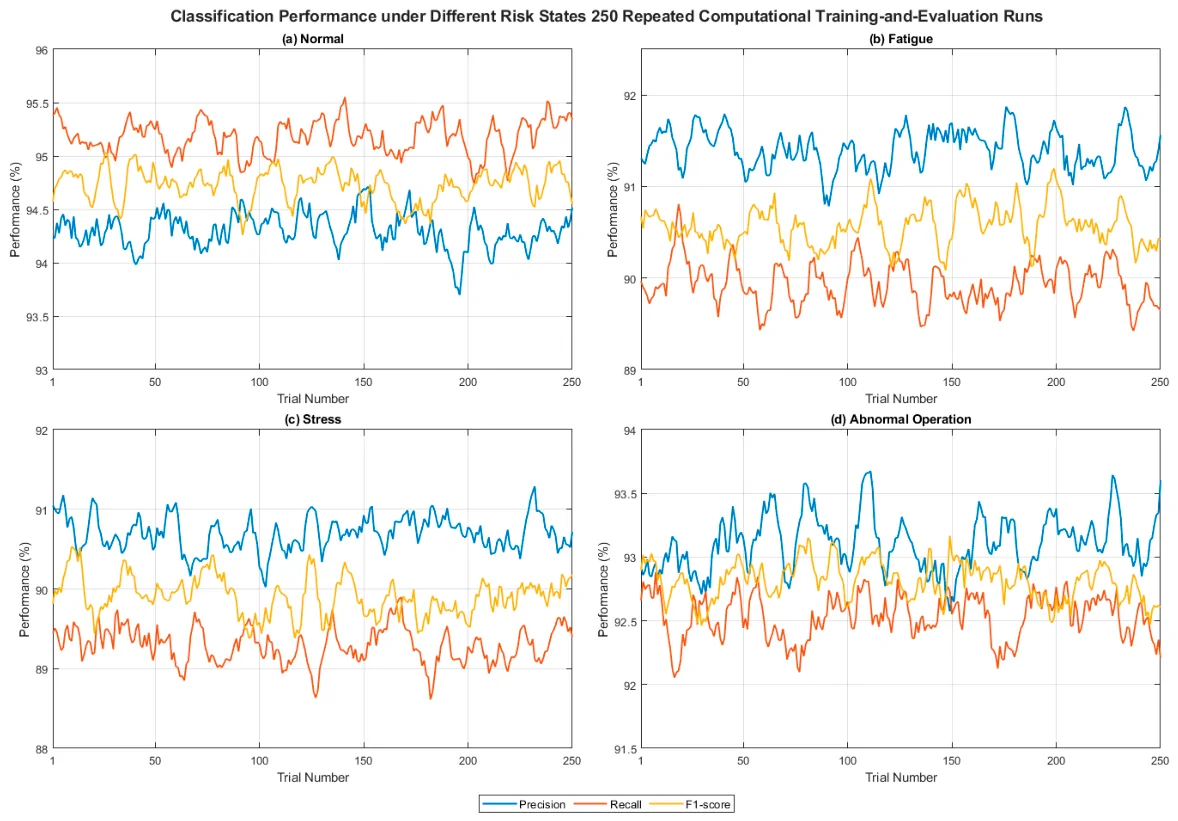
Classification performance curves of the proposed method under different risk states over 250 repeated computational training-and-evaluation runs.

To further compare recognition differences of the proposed method under different risk states, 250 repeated computational training-and-evaluation runs are conducted separately for normal, fatigue, stress, and abnormal-operation states, and the Precision, Recall, and F1-score corresponding to each trial are calculated. Figure 5 uses a 2 × 2 subplot format to show variations in the three metrics with the number of trials for the four states. The vertical-axis ranges of the individual subplots are set according to the actual distributions of the corresponding curves to more clearly reflect the recognition levels and fluctuation characteristics of the model under different risk states.

As shown in Figure 5, the proposed method maintained relatively high Precision, Recall, and F1-score values across all four states over the repeated computational runs. The normal state achieved the highest average Precision, Recall, and F1-score (94.28%, 95.17%, and 94.72%, respectively), indicating that normal samples were relatively well distinguished from risk-state samples. The abnormal-operation state also achieved high values of 93.15%, 92.47%, and 92.81%, respectively.

Fatigue and stress produced comparatively lower and closer results. For fatigue, Precision, Recall, and F1-score were 91.36%, 89.84%, and 90.59%, respectively; for stress, the corresponding values were 90.74%, 89.21%, and 89.97%. The lower Recall for stress suggests residual confusion with fatigue and abnormal-operation states, which may be related to overlapping physiological–behavioral patterns and differences in task load or event intensity. Future work should incorporate contextual information, such as alarm type, task complexity, continuous-operation duration, and task-switching frequency, to improve discrimination between fatigue and stress.

The AUC values exceeded 94% for all four states, reaching 97.36% for normal operation and 96.48% for abnormal operation, compared with 95.12% for fatigue and 94.63% for stress. Overall, Figure 5 indicates clearer class separation for normal and abnormal-operation states, whereas fatigue and stress remain more difficult to distinguish. These results support the effectiveness of the proposed multimodal temporal classification framework under the evaluated experimental conditions.

### 5.3. Ablation Study

To verify the specific contributions of each key module in the proposed method to human-factor risk-recognition performance, this manuscript further conducts ablation experiments. Taking the complete model as the benchmark, the physiological modality, behavioral modality, physiological–behavioral coupling module, attention mechanism, and temporal-modeling module are removed in sequence, and comparisons are conducted under the same data partitioning, training parameters, and evaluation metrics. Each group of experiments is repeated 250 times, and the results are reported in the form of mean ± standard deviation.

The settings of the different ablation models are as follows:

w/o Physiological modality: the physiological-feature branch is removed, and only behavioral features are retained;

w/o Behavioral modality: the behavioral-feature branch is removed, and only physiological features are retained;

w/o Coupling module: dual-modal input is retained, but the physiological–behavioral coupling term is removed, and only conventional fusion is adopted;

w/o Attention module: the attention mechanism is removed, and fixed-weight or direct-concatenation fusion is adopted;

w/o Temporal modeling: LSTM temporal modeling is removed, and classification is performed based only on a single time window;

Full model: the complete model retains physiological features, behavioral features, coupling modeling, the attention mechanism, and temporal modeling.

The ablation results are shown in Table 7. It should be noted that the results in the table represent the mean ± standard deviation of 250 repeated computational training-and-evaluation runs.

As can be seen from Table 7, removing any key module leads to a decline in model performance, indicating that all components have positive effects on human-factor risk recognition. Among them, after removing the physiological or behavioral modality, model Accuracy decreases to 79.84% and 80.92%, respectively, representing the most obvious decreases. This indicates that physiological and behavioral information have strong complementarity and that a single modality has difficulty completely representing changes in the operator’s internal state and external operating performance under complex task environments.

After removing the temporal-modeling module, model Accuracy decreases from 92.16% to 87.46%, and Macro-F1 decreases from 90.86% to 85.94%, while FAR and MDR increase to 7.61% and 10.37%, respectively. This indicates that human-factor risks such as fatigue, stress, and abnormal operation have obvious temporal continuity and accumulation characteristics, and using only a single time window is insufficient to fully reflect the risk-formation and evolution process.

When the physiological–behavioral coupling module is removed, model Accuracy and Macro-F1 decrease to 88.21% and 86.73%, respectively. This result indicates that operator risk is not determined independently by physiological deviation or behavioral abnormality, but is affected by their joint action. If only conventional fusion is conducted without characterizing the relationship between physiological-state changes and operating-behavior abnormalities, the model’s discriminative capability for complex risk states is significantly weakened.

After removing the attention mechanism, model Accuracy and Macro-F1 become 89.03% and 87.56%, respectively, decreasing by 3.13 and 3.30 percentage points compared with the complete model. Meanwhile, FAR and MDR increase by 2.00 and 2.47 percentage points, respectively. This indicates that the importance of key features differs under different risk states, and fixed-weight or direct-concatenation methods have difficulty fully highlighting physiological and behavioral information related to the current state. The attention mechanism can dynamically adjust modality and feature contributions according to risk type, thereby enhancing model recognition capability and warning reliability.

In terms of the contribution degree of different modules, temporal modeling, physiological–behavioral coupling, and the attention mechanism all play important roles in improving model performance. Among them, removing temporal modeling causes the most obvious performance decline, indicating that dynamic evolution information of risk states is an important basis for human-factor risk recognition. Removing the coupling module and attention mechanism also causes obvious performance degradation, further verifying the necessity of physiological–behavioral association modeling and dynamic feature weighting.

**Figure 6:**
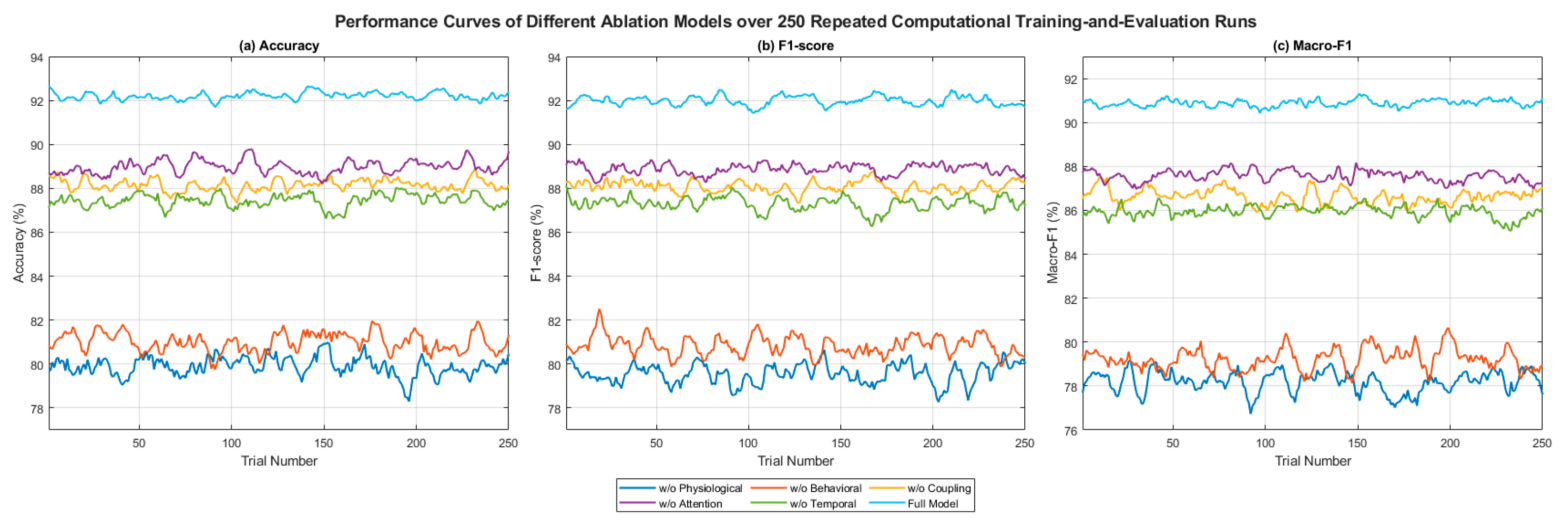
Performance curves of different ablation models over 250 repeated computational training-and-evaluation runs.

To further analyze model performance stability after removing different modules, 250 repeated computational training-and-evaluation runs are conducted for each ablation model, and variations in Accuracy, F1-score, and Macro-F1 are recorded. Figure 6 takes the trial number as the horizontal axis and the evaluation metric value as the vertical axis, showing the performance variation curves of the different ablation models during repeated trials.

All results in Table 7 are reported as the mean ± standard deviation over 250 repeated computational training-and-evaluation runs. The participant-independent data partition, preprocessing procedure, feature configuration, model hyperparameters, and held-out test set were fixed across runs. Only the random seed used for model initialization and mini-batch ordering was changed. Therefore, the reported standard deviations reflect the stability of stochastic model optimization rather than variation across independent participant experiments.

As can be seen in Figure 6, the Accuracy, F1-score, and Macro-F1 curves of the complete model remain at the highest overall levels over the 250 repeated computational training-and-evaluation runs and have the smallest fluctuations, indicating that the model not only has higher average recognition performance but also better repeated-trial stability. In comparison, after removing the physiological modality or behavioral modality, all three metric curves decrease significantly, while the fluctuation amplitudes increase, indicating that a single modality has difficulty stably and completely representing operator human-factor risk states.

Among the functional-module ablation results, removing the temporal-modeling module results in the most obvious performance decline, with the Accuracy, F1-score, and Macro-F1 curves generally lower than those obtained after removing the coupling module or the attention mechanism. This indicates that information from consecutive time windows plays an important role in characterizing dynamic characteristics such as fatigue accumulation, stress triggering, and continuous evolution of abnormal operation. After removing the physiological–behavioral coupling module, the metrics also decrease significantly, indicating that explicitly modeling the association between physiological-state changes and behavioral abnormalities can enhance the discriminative capability of risk features.

After removing the attention mechanism, although model performance remains better than that obtained after removing temporal modeling or the coupling module, all three metric curves are lower than those of the complete model, and experimental fluctuations increase. This indicates that the attention mechanism can dynamically adjust the contributions of physiological and behavioral features according to different risk states, thereby reducing interference from redundant information and improving the stability of model outputs.

Overall, Figure 5 further demonstrates, from the perspective of repeated trials, that the physiological modality, behavioral modality, temporal modeling, physiological–behavioral coupling mechanism, and attention mechanism all have positive effects on model performance and stability. Among them, the complete model exhibits higher average levels and smaller fluctuations for all metrics.

**Figure 7:**
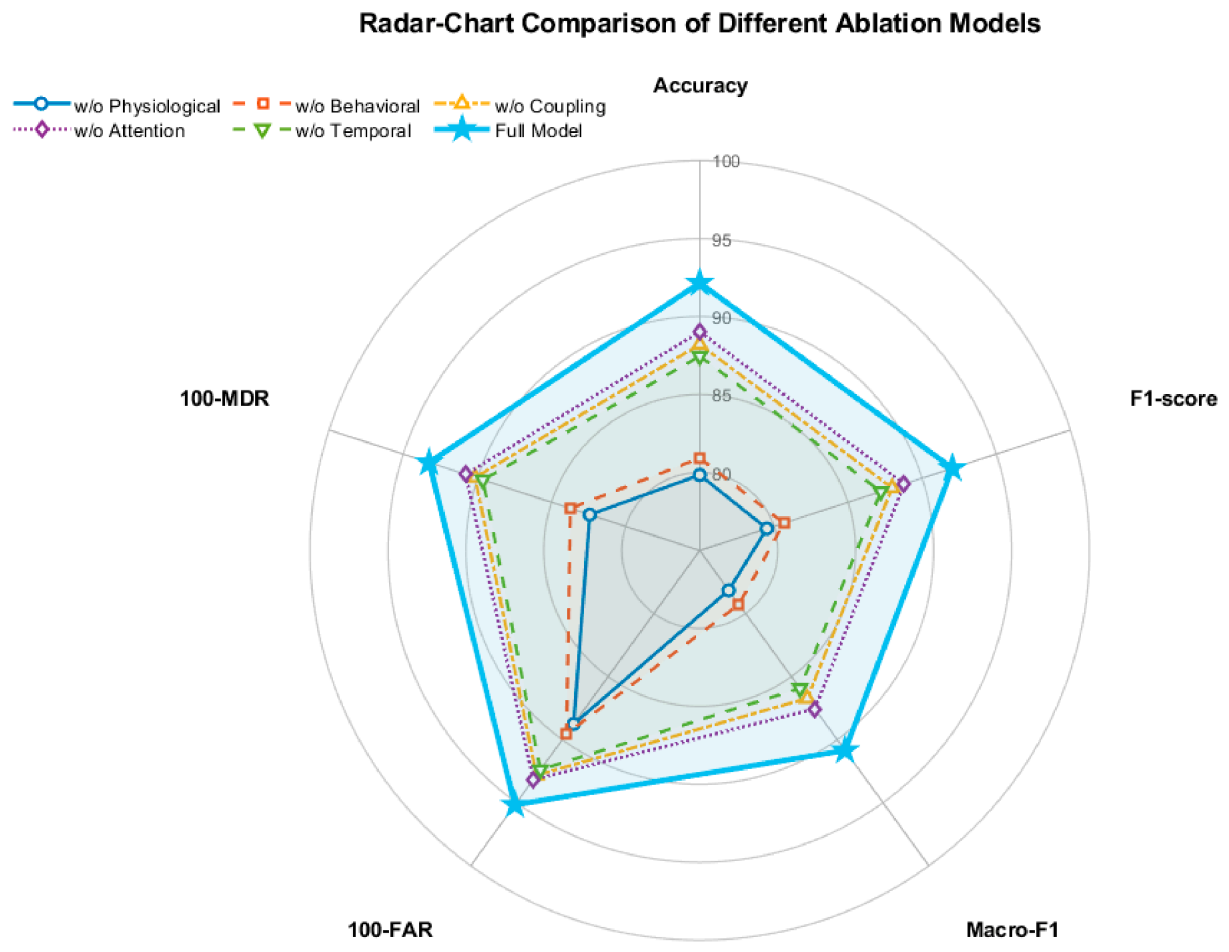
Radar-Chart comparison of different ablation models.

To more intuitively compare the influence of different component modules on the overall model performance, this manuscript uses a radar chart to visualize the comprehensive performance of the ablation models, as shown in Figure 7. The radar chart selects Accuracy, F1-score, Macro-F1, 100-FAR, and 100-MDR as evaluation dimensions. The first three metrics reflect model classification performance, while the latter two reflect model warning reliability. By converting FAR and MDR into positive indicators, all dimensions maintain a consistent direction; that is, a larger value indicates better model performance.

As can be seen in Figure 7, the complete model exhibits the largest envelope area across all evaluation dimensions, indicating that it achieves the best comprehensive performance in classification accuracy, class balance, and warning reliability. In comparison, after any key module is removed, the radar-chart area shrinks to different degrees, indicating that all components have positive effects on model performance improvement.

Among them, after removing the physiological or behavioral modality, the overall contraction of the radar chart is the most obvious, particularly in the Accuracy, F1-score, and Macro-F1 dimensions, while the values of 100-FAR and 100-MDR also decrease significantly. This indicates that physiological and behavioral information have strong complementarity in operator human-factor risk recognition. The absence of either modality weakens the completeness of state representation and reduces the reliability of risk warnings.

For the functional modules, after removing the temporal-modeling module, the radar chart shows the most obvious contraction in the Accuracy, Macro-F1, and 100-MDR dimensions, indicating that temporal information plays an important role in characterizing dynamic features such as fatigue accumulation, sudden stress, and continuous evolution of abnormal operation. After removing the physiological–behavioral coupling module, the model also shows obvious degradation in the F1-score and Macro-F1 dimensions, indicating that explicitly modeling the association between physiological-state changes and behavioral abnormalities helps form more discriminative risk representations.

In addition, after removing the attention mechanism, the degradation of the model across the dimensions is slightly lower than that caused by removing temporal modeling or the coupling module, but the envelope still shrinks considerably. This indicates that the attention mechanism can dynamically highlight physiological and behavioral features with higher discriminative value according to different risk states, thereby reducing interference from irrelevant information and improving the model’s adaptability to complex risk states.

Overall, Figure 6 further verifies the important roles of the physiological modality, behavioral modality, temporal modeling, physiological–behavioral coupling mechanism, and attention mechanism in the proposed method. Through multimodal information complementarity, dynamic temporal modeling, and adaptive weighting of key features, the complete model achieves the best comprehensive recognition performance and warning reliability.

### 5.4. Interpretability Analysis

To further analyze the decision basis of the proposed model during human-factor risk recognition, this manuscript conducts interpretability analysis from three aspects: modality-level attention allocation, feature contribution, and physiological–behavioral coupling relationships. Unlike a black-box model that only outputs risk categories, the proposed method can reflect the relative contributions of physiological and behavioral modalities under different states through attention weights and further identify key features that have relatively high impacts on the discrimination of normal, fatigue, stress, and abnormal-operation states.

(1) Modality-Level Attention Analysis

The physiological-modality and behavioral-modality attention weights corresponding to different risk states in the test set are statistically analyzed. Since the attention weights of the two modalities are normalized, their sum is 1. The average modality attention weights under different risk states are shown in Table 8.

As can be seen from Table 8, the contributions of the two modalities differ significantly under different risk states. Under the normal state, the average weights of the physiological and behavioral modalities are 0.48 and 0.52, respectively, which are relatively close. This indicates that when the operator is in a stable state, neither physiological indicators nor operating behaviors exhibit obvious abnormalities, and the model needs to comprehensively use both types of information for judgment.

Under fatigue, the physiological-modality weight increases to 0.61, higher than the 0.39 of the behavioral modality. This indicates that fatigue recognition relies more on internal physiological signals such as heart rate variability, respiratory rhythm, and blood-oxygen changes. Fatigue usually accumulates gradually with continuous operation time, and physiological changes may occur earlier than obvious behavioral abnormalities. Therefore, the model assigns a higher weight to physiological information under this state.

Under stress, the physiological-modality weight is the highest, reaching 0.66. Sudden alarms, task conflicts, or complex task switching may cause increased heart rate, accelerated respiration, and enhanced electrodermal activity within a short period. Therefore, physiological features have relatively high discriminative value for stress recognition. The behavioral modality still accounts for a weight of 0.34, indicating that behavioral changes such as response delay, manipulation frequency, and repeated corrections are also important bases for stress recognition.

Under abnormal operation, the behavioral-modality weight increases to 0.63, significantly higher than the 0.37 of the physiological modality. This is because abnormal operation is usually directly manifested as external behavioral abnormalities such as trajectory deviation, erroneous commands, untimely response, and task interruption. Although physiological changes can provide auxiliary information, behavioral data are more directly associated with abnormal-operation outcomes.

**Figure 8:**
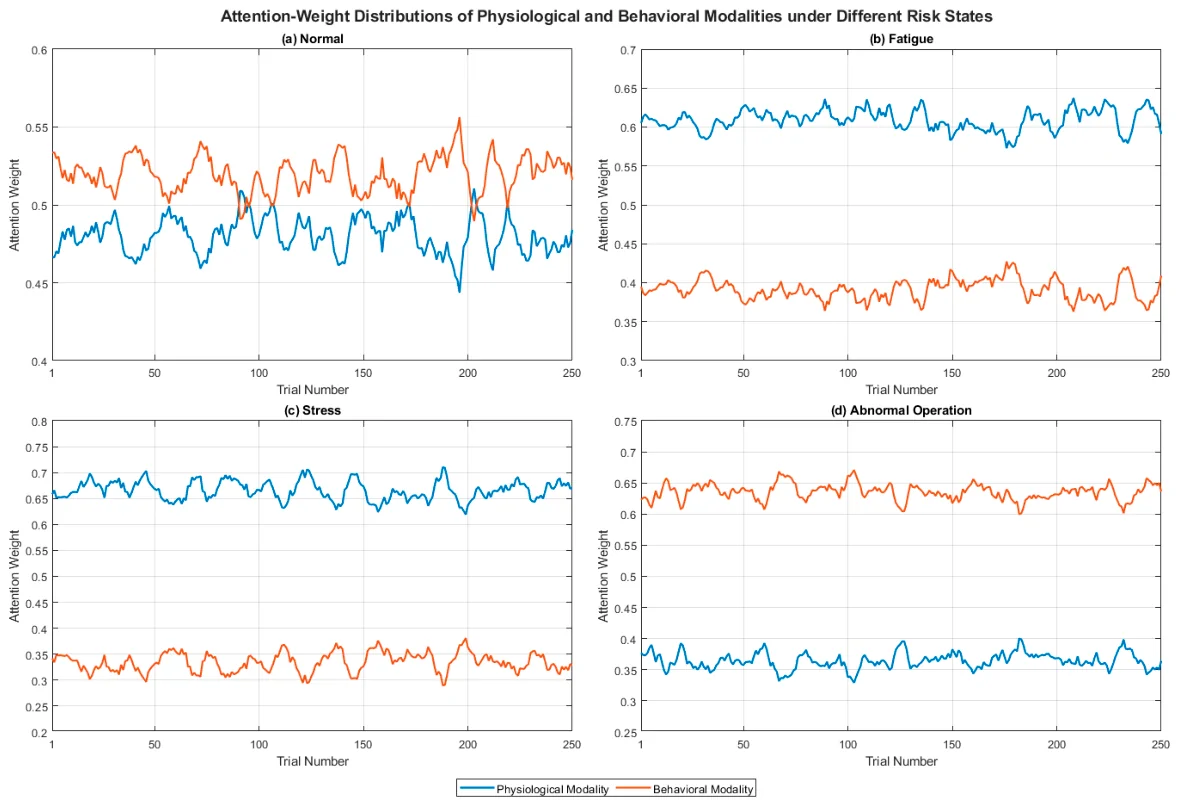
Distribution of physiological and behavioral modality attention weights under different risk states.

To more intuitively show the differences in modality contributions under different risk states, this manuscript plots the comparison results of the attention weights of physiological and behavioral modalities, as shown in Figure 8.

Figure 8 shows that the attention mechanism does not adopt fixed weights for the physiological and behavioral modalities, but dynamically adjusts the contributions of the two types of information according to the operator’s current state. This result is basically consistent with the actual formation mechanisms of fatigue, stress, and abnormal operation, thereby verifying the rationality of the attention-allocation results at the model level.

(2) Feature-Level Contribution Analysis

On the basis of the modality-level analysis, this manuscript further calculates the contributions of individual physiological and behavioral features to the prediction results under different risk states. The interpretability analysis in this study is conducted at the modality level. The implemented attention module produces adaptive weights for the physiological and behavioral representations before fusion, indicating their relative contributions to the predicted risk state. These weights are not interpreted as importance scores for individual input features. A validated feature-level attribution analysis was beyond the scope of the present pilot study and will be investigated in future work.

**Figure 9:**
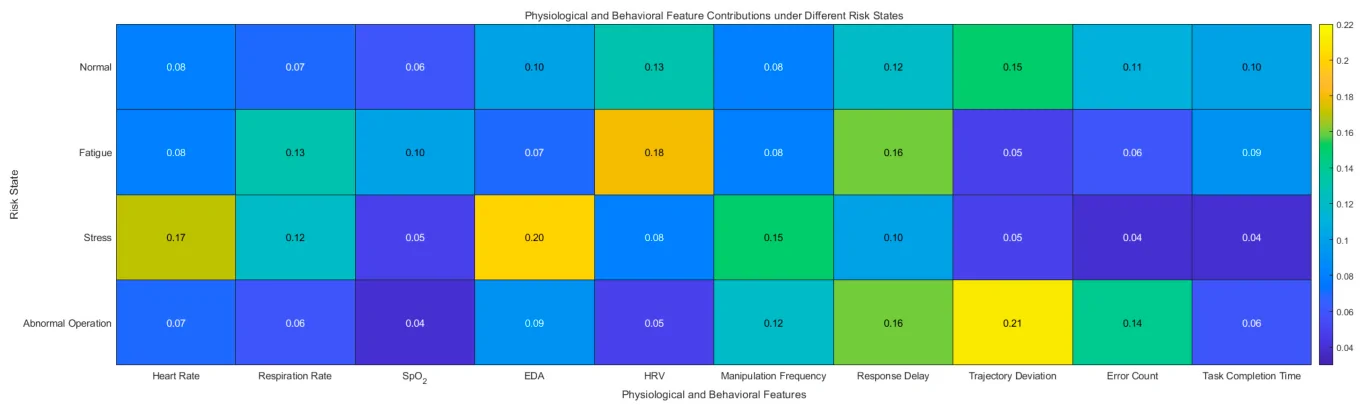
Heatmap of physiological and behavioral feature contributions under different risk states.

To show the overall distribution of feature contributions under different risk states, this manuscript plots a feature-contribution heatmap, as shown in Figure 9.

As can be seen in Figure 9, different risk states exhibit differentiated feature-contribution patterns. Fatigue has relatively high contributions from features such as HRV, response delay, and task-completion time; stress is mainly concentrated on EDA, heart rate, respiratory rate, and manipulation frequency; abnormal operation is mainly concentrated on trajectory deviation, number of erroneous operations, and response delay. These differences indicate that the proposed model can select corresponding key features according to different risk states rather than adopting the same discrimination rule for all states.

(3) Physiological–Behavioral Coupling Analysis

In addition to individual feature contributions, this manuscript further analyzes the coupling relationships between physiological changes and behavioral abnormalities. The coupling weight reflects the joint influence on the risk-recognition result when a change in a certain physiological feature and an abnormality in a certain behavioral feature occur simultaneously. A higher coupling weight indicates a stronger risk association between the two types of features.

The analysis results show that the relatively strong coupling relationships under fatigue mainly include HRV and response delay, respiratory rate and task-completion time, and blood-oxygen changes and number of operating errors. These associations indicate that after internal physiological fatigue gradually accumulates, it may further manifest as decreased response speed, reduced task-execution efficiency, and increased erroneous operations.

Under stress, relatively strong coupling relationships are mainly manifested between electrodermal activity and manipulation frequency, heart rate and repeated correction behavior, and respiratory rate and response delay. Physiological arousal caused by sudden stimuli may cause operators to accelerate their operating rhythm, make frequent corrections, or exhibit fluctuations in response behavior, thereby forming typical physiological–behavioral joint stress patterns.

Under abnormal operation, certain coupling relationships still exist between trajectory deviation and the number of erroneous operations on the one hand and physiological features such as electrodermal activity and heart rate on the other. However, the overall coupling strength is lower than the direct contribution of the behavioral features themselves. This indicates that abnormal-operation risk is mainly manifested through external behavioral outcomes, while physiological signals are used more as auxiliary information to determine whether the abnormality is caused by nervousness, fatigue, or state instability.

The above results provide model-based evidence that the physiological–behavioral coupling representation is associated with improved risk-classification performance relative to simple feature concatenation. Unlike simple feature concatenation, coupling modeling can further describe the association process through which changes in an operator’s internal state evolve into external behavioral abnormalities, thereby providing a clearer explanation for the risk-classification results.

(4) Discussion of Interpretability Results

By considering the modality-attention and physiological–behavioral coupling analyses, the proposed method shows different relative contributions of physiological and behavioral information across risk states. These model-derived patterns are consistent with the expected complementary roles of internal physiological changes and external behavioral performance in human-factor risk assessment.

The dynamic variations in attention weights indicate that the model can adaptively adjust the importance of physiological and behavioral information according to different risk states. The dynamic variations in modality-attention weights indicate that the model can adaptively adjust the relative importance of physiological and behavioral representations according to the input condition. Coupling-relationship analysis further describes the joint association between internal physiological changes and external behavioral abnormalities. These results provide modality-level information for interpreting model outputs.

Overall, the modality-level attention and coupling-analysis results are consistent with the intended physiological–behavioral rationale of the proposed framework and support its potential engineering value. However, these model-derived results should not be interpreted as causal evidence or as independent biological validation.

## 6. Discussion

This manuscript proposes a bio-inspired physiological–behavioral fusion risk assessment method for human-factor safety issues in imported unmanned intelligent system operation scenarios at ports. Starting from human stress responses, neural feedback, and selective attention mechanisms, the proposed method incorporates changes in the operator’s physiological state, abnormalities in behavioral responses, and their temporal evolution processes into a unified modeling framework. The experimental results show that the proposed method demonstrates good effectiveness in terms of overall recognition performance, recognition under different risk states, ablation experiments, and interpretability analysis. Compared with traditional static classification methods, single-modal recognition methods, and conventional temporal fusion models, the proposed method can make fuller use of the complementary relationship between physiological and behavioral information, while improving the stability of human-factor risk recognition and reducing false alarm and missed detection rates. The biological concepts in this study serve as design motivations for the overall architecture; the attention mechanism, coupling representation, temporal classifier, and optimization procedure are conventional engineering and machine-learning components.

First, the joint modeling of physiological and behavioral information is an important basis for improving operator-state recognition performance. Physiological signals can reflect fatigue, stress, and changes in physiological load at an earlier stage. In particular, indicators such as heart rate, respiratory rate, electrodermal activity, and heart rate variability are highly sensitive to the operator’s internal state. However, physiological signals are easily affected by individual differences, motion artifacts, environmental changes, and sensor noise, and their independent use may lead to unstable state discrimination. Behavioral features can directly reflect the operator’s response capability, operating stability, and task-execution quality. Response delay, trajectory deviation, and the number of erroneous operations are relatively directly associated with actual operational risks, but their abnormal manifestations usually occur later than internal physiological changes. In the experiments, the multimodal models significantly outperform the two single-modal models, indicating that physiological and behavioral features can jointly describe operator risk from the perspectives of internal state and external performance, thereby improving the completeness of risk representation.

Second, temporal modeling plays an important role in human-factor risk recognition. Fatigue, stress, and abnormal operation are not completely independent instantaneous states, but have obvious temporal continuity, accumulation, and state-transition characteristics. Fatigue usually develops gradually as continuous operation time increases; stress may be rapidly triggered by sudden alarms, and abnormal operation may be manifested as delayed response, frequent corrections, and trajectory deviation over multiple consecutive time windows. The experimental results show that LSTM-based fusion significantly outperforms traditional static classification models. In the ablation experiments, after the temporal-modeling module is removed, the Accuracy, F1-score, and Macro-F1 of the model all decrease significantly, while the false alarm and missed detection rates increase. This indicates that relying only on statistical features within a single time window makes it difficult to fully reflect the dynamic evolution of operator risk states, whereas modeling consecutive time windows can reduce the influence of instantaneous noise on recognition results and enhance the recognition capability for persistent and cumulative risks.

Third, physiological–behavioral coupling modeling further improves the discriminative capability for complex risk states. Traditional multimodal methods usually adopt feature concatenation or fixed-weight fusion. Although such methods can increase the amount of input information, they do not explicitly describe the association process between internal physiological changes and external behavioral abnormalities. In this manuscript, a coupling term is used to characterize the mechanism through which physiological-state deviation and behavioral-performance abnormalities jointly contribute to risk formation. The ablation results show that model performance decreases significantly after the coupling module is removed, indicating that operator risk is not independently determined by a single physiological abnormality or a single behavioral abnormality, but is affected by the joint changes of the two.

An increase in heart rate may be caused by short-term task pressure, and response delay may also result from increased task complexity. However, when abnormal physiological indicators occur simultaneously with response delay, trajectory deviation, and erroneous operations, the probability that the operator is in a high-risk state increases significantly. Therefore, coupling modeling helps reduce misjudgments caused by a single abnormal signal and forms a more discriminative comprehensive risk representation.

Fourth, the modality-level attention mechanism improves the adaptability of multimodal fusion under different input conditions. Physiological and behavioral information may provide different levels of predictive contribution across risk states. The implemented attention module therefore adaptively weights the two modality representations before fusion, whereas the temporal classifier captures sequential dependence from the fused sequence. Both the attention mechanism and the LSTM are conventional machine-learning design choices.

The observed modality-weight patterns are consistent with the expected complementary roles of physiological and behavioral information in human-factor risk assessment. However, these model-derived weights should not be interpreted as causal evidence, independent biological validation, or proof that the model reproduces biological selective attention.

From the class-specific recognition results, the normal and abnormal operation states have relatively clear decision boundaries, whereas there is still a certain degree of confusion between fatigue and stress. The normal state is usually manifested by relatively stable physiological indicators and behavioral performance, whereas abnormal operation is often accompanied by obvious trajectory deviation, erroneous commands, and task interruption; therefore, these two states are relatively easy to distinguish.

In comparison, fatigue and stress overlap in some physiological and behavioral features. Both may involve changes in heart rate variability, abnormal respiratory rhythm, response delay, and decreased operating stability. The difference is that fatigue usually has a relatively obvious accumulation process, whereas stress is more easily affected by sudden events and task contexts. In the future, task-context information such as continuous operation duration, alarm type, task complexity, task-switching frequency, and environmental disturbance intensity can be further introduced to enhance the model’s capability to distinguish between fatigue and stress.

From an engineering-application perspective, the proposed method has certain potential for port-supervision applications. For imported unmanned intelligent systems such as unmanned aerial vehicles, unmanned ground vehicles, and unmanned surface vehicles, operators usually undertake key responsibilities such as task planning, status monitoring, abnormal takeover, and safety handling.

Based on the human-factor risk assessment results obtained through physiological–behavioral fusion, a graded warning and intervention mechanism can be further constructed. When the model recognizes fatigue risk, it can prompt the operator to rest, reduce continuous operation duration, or adjust the task load. When a stress state is recognized, the number of concurrent tasks can be reduced, operation confirmation can be increased, or decision-making assistance can be provided. When abnormal operation or a high-risk state is detected, manual review, task suspension, or safety takeover can be triggered. By combining risk-recognition results with operation-management procedures, the influence of abnormal operator states on the operational safety of unmanned intelligent systems can be reduced to a certain extent.

Although the proposed method achieves good experimental results, several limitations remain.

First, the current study mainly constructs experimental scenarios around a limited number of unmanned intelligent system operation tasks. Different equipment platforms, task modes, and port environments may have different human-factor risk characteristics. Future work should expand the coverage of unmanned aerial vehicles, unmanned ground vehicles, unmanned surface vehicles, and multi-device collaborative tasks to verify the cross-platform adaptability of the model.

Second, obvious individual differences exist among operators, including physiological baselines, operating experience, stress tolerance, and behavioral habits. Although baseline deviation and standardization methods are adopted in this manuscript to reduce the influence of individual differences, individual-adaptive and few-shot calibration methods still need to be investigated for large-scale applications.

Third, the existing physiological-signal acquisition process relies on wearable or contact-based sensing devices and may be affected by wearing comfort, motion interference, and missing data. In the future, multi-source physiological-state acquisition methods based on non-contact vision, seat sensors, steering-wheel sensors, or environmental sensing devices can be further investigated to improve system usability during long-term operation.

Fourth, the interpretability analysis in this manuscript is limited to modality-level attention weights and coupling analysis. These model-derived results reflect the relative weighting of the two modalities, but attention weights are not equivalent to causal explanations or biological validation. Future work should use validated feature-attribution and causal-analysis methods, such as SHAP, counterfactual analysis, and controlled physiological experiments, to investigate individual-feature effects and underlying mechanisms.

Fifth, the current study mainly focuses on model-recognition performance and does not fully discuss the effects of risk-warning thresholds, intervention timing, and intervention strategies on subsequent operator states. In practical applications, overly sensitive warnings may increase operator burden, whereas overly conservative thresholds may result in delayed risk detection. Therefore, future work needs to investigate dynamic warning thresholds and human–machine collaborative intervention strategies according to different risk levels, task importance, and handling costs, and evaluate the effects of intervention measures on risk-state mitigation and task-completion quality through long-term experiments.

An additional limitation is that the present study did not evaluate end-to-end online performance. The reported experiments focused on offline risk-state classification and repeated computational stability; inference latency, preprocessing latency, warning-generation latency, hardware configuration, computational complexity, memory consumption, and multi-operator concurrent-processing capability were not measured. Therefore, the current results should not be interpreted as demonstrating real-time deployment capability. Future work will evaluate the complete processing pipeline, including signal acquisition, synchronization, preprocessing, feature construction, model inference, and warning generation, under specified hardware and operational conditions before practical online deployment is claimed.

An additional limitation concerns label independence and partial scenario–class association. The risk labels in this study were operational labels derived from predefined task criteria and documented task events, rather than clinical ground truth established using validated self-report scales or independent physiological diagnosis. In particular, continuous-operation periods, sudden-alarm handling, and safety-relevant task events were used to identify candidate fatigue, stress, and abnormal-operation states, respectively. Although scenario identity was not included as an explicit model-input feature and was not the sole basis for labeling, task context may still influence the physiological and behavioral patterns available to the classifier. The participant-level split prevents operator-level data leakage but does not eliminate this potential scenario–class confounding. Therefore, the present results should be interpreted as pilot evidence for operational risk-state classification under the examined port-operation conditions, rather than as scenario-independent recognition of latent physiological states. Future studies should collect each risk state across multiple task scenarios and conduct cross-scenario and within-scenario validation.

Overall, the results of this manuscript indicate that mapping human stress responses, neural feedback, and selective attention mechanisms into the operator risk-recognition process can provide an effective approach for human-factor safety assessment under complex human–machine collaborative scenarios. Physiological–behavioral dual-modal input improves the completeness of state representation, temporal modeling enhances the capability for risk-evolution analysis, the coupling mechanism characterizes the joint influence between internal states and external behavior, and the attention mechanism provides modality-level adaptive weighting and supports interpretation of the model’s multimodal fusion process. The proposed method provides a methodological basis for dynamic human-factor risk recognition, graded warning, and safety intervention for operators of imported unmanned intelligent systems at ports.

## 7. Conclusions

This manuscript addresses human-factor safety risks, such as fatigue, stress, delayed response, and abnormal operation, during the operation of imported unmanned intelligent systems at ports, including unmanned aerial vehicles, unmanned ground vehicles, and unmanned surface vehicles, and proposes a bio-inspired physiological–behavioral fusion risk assessment method inspired by human stress responses, neural feedback, and selective attention mechanisms. The proposed method incorporates changes in operator physiological states, operating-behavior performance, and their temporal evolution into a unified modeling framework. Through dual-branch physiological and behavioral feature extraction, physiological–behavioral coupling modeling, attention-weighted fusion, and temporal risk classification, the method realizes dynamic recognition and classified assessment of normal, fatigue, stress, and abnormal-operation states. The biological concepts motivate the organization of the framework, whereas the implemented attention, coupling, temporal-classification, and optimization components are conventional machine-learning choices and are not presented as direct biological or neural models.

The experimental results show that the proposed method outperforms traditional machine-learning models, single-modal models, the direct feature-concatenation model, and the conventional temporal fusion model in terms of overall recognition performance, warning reliability, and repeated-trial stability. Over 250 repeated computational training-and-evaluation runs, the Accuracy, Precision, Recall, F1-score, and Macro-F1 of the proposed method reach 92.16%, 92.43%, 91.68%, 92.04%, and 90.86%, respectively, while FAR and MDR are reduced to 4.83% and 6.74%, respectively.

Compared with the LSTM-based fusion model, the proposed method improves Accuracy, F1-score, and Macro-F1 by 2.60, 2.70, and 2.69 percentage points, respectively, and reduces FAR and MDR by 1.71 and 2.22 percentage points, respectively, indicating that physiological–behavioral coupling and the attention mechanism can further improve multimodal temporal risk-recognition capability.

The experimental results under different risk states further show that the proposed method has relatively clear decision boundaries for normal and abnormal-operation states and also maintains relatively high recognition performance for fatigue and stress. There is still a certain degree of confusion between fatigue and stress, mainly because the two states partially overlap in features such as heart rate variability, respiratory rhythm, response delay, and operating stability.

The ablation experiments show that the physiological modality, behavioral modality, temporal modeling, physiological–behavioral coupling mechanism, and attention mechanism all make important contributions to model performance. Among them, removing either modality or the temporal-modeling module causes a relatively obvious performance decline.

The interpretability analysis shows that different risk states are associated with different relative weights of the physiological and behavioral modalities. The observed modality-weight patterns are consistent with expected physiological–behavioral characteristics of fatigue, stress, and abnormal operation; however, they should not be interpreted as causal or independently validated biological mechanisms.

Overall, the proposed method can simultaneously utilize internal physiological changes and external operating behaviors of operators, thereby improving the completeness, dynamics, and interpretability of human-factor risk-state representation under complex unmanned intelligent system operation scenarios. Within the examined port-operation tasks, the proposed method provides preliminary evidence for physiological–behavioral fusion in operator risk-state monitoring and classification. Its applicability to independent task scenarios and broader port-operation conditions requires further validation using cross-scenario experimental protocols.

Future research will further expand the number of operators and types of unmanned intelligent systems and cover more port task modes and complex environmental conditions; investigate adaptive modeling and few-shot calibration methods for individual differences; introduce contextual information such as task load, alarm type, and environmental complexity to improve the discrimination capability between fatigue and stress; and combine non-contact physiological sensing, model light weighting, and edge-computing deployment, and evaluate their effects on latency, long-term stability, and engineering applicability in practical port-supervision scenarios.

## Figures and Tables

**Table 1:** Mapping relationships between bio-inspired mechanisms and the operator human-factor risk assessment model.

Bio-Inspired Mechanism	Meaning in the Biological System	Engineering Mapping in Operator Risk Assessment
External stimulus perception	Perception of task pressure, environmental changes, and sudden stimuli	Task load, abnormal alarms, environmental complexity, and continuous operation duration
Autonomic nervous response	Physiological changes such as heart rate, respiration, and electrodermal activity	Physiological features such as heart rate, respiratory rate, blood oxygen, electrodermal activity, and heart rate variability
Behavioral response output	Response speed, movement stability, and behavioral adjustment	Manipulation frequency, response delay, trajectory deviation, number of erroneous operations, and task-completion time
Multi-source information integration	Comprehensive processing of physiological and behavioral feedback by the brain	Fusion representation of physiological and behavioral features
Selective attention	Dynamic allocation of attention weights according to information importance	Adaptive feature weighting under the attention mechanism
Neural feedback regulation	Behavioral correction and state regulation according to the risk state	Risk-level output, warning prompts, and intervention feedback

**Table 2:** Typical operation scenarios and target risk states.

Experimental Scenario	Main Task Characteristics	Target State
Normal operation	Routine monitoring and standardized operation without sudden events	Normal
Long-duration continuous operation	Continuous monitoring, repetitive manipulation, and prolonged task duration	Fatigue
Sudden abnormal-alarm handling	Random alarms, time-limited response, abnormality judgment, and handling	Stress
Complex multi-task collaboration	Parallel multi-device operation, frequent switching, and task conflicts	Abnormal operation or high risk

**Table 3:** Evaluation metrics for the human-factor risk recognition model.

Metric Category	Specific Metric	Evaluation Purpose
Overall performance	Accuracy	Measures the overall classification accuracy
Class performance	Precision, Recall, F1-score	Measures the recognition capability for each risk state
Balanced performance	Macro-F1, Macro-AUC	Reduces the influence of class imbalance
Warning reliability	False Alarm Rate, Missed Detection Rate	Evaluates false alarms and missed detections

**Table 4:** Overall performance comparison of human-factor risk recognition models over 250 repeated computational training-and-evaluation runs.

Method	Accuracy (%)	Precision (%)	Recall (%)	F1-Score (%)	Macro-F1 (%)	FAR (%)	MDR (%)
SVM	78.63 ± 1.25	79.14 ± 1.28	77.86 ± 1.34	78.27 ± 1.31	76.94 ± 1.38	11.83 ± 0.92	18.46 ± 1.25
Random Forest	82.47 ± 1.08	82.73 ± 1.11	81.96 ± 1.16	82.18 ± 1.12	80.84 ± 1.19	9.64 ± 0.78	14.17 ± 1.06
XGBoost	84.76 ± 0.96	85.08 ± 0.99	84.13 ± 1.03	84.57 ± 0.98	83.46 ± 1.05	8.73 ± 0.69	12.64 ± 0.93
Physiological-only model	80.92 ± 1.18	81.46 ± 1.21	80.08 ± 1.25	80.67 ± 1.20	79.28 ± 1.27	10.46 ± 0.86	16.27 ± 1.16
Behavioral-only model	79.84 ± 1.22	80.57 ± 1.25	78.96 ± 1.30	79.53 ± 1.26	78.16 ± 1.31	11.24 ± 0.89	17.58 ± 1.21
Direct feature concatenation	86.37 ± 0.82	86.84 ± 0.86	85.76 ± 0.89	86.18 ± 0.85	84.96 ± 0.91	7.94 ± 0.61	10.86 ± 0.78
LSTM-based fusion	89.56 ± 0.68	89.93 ± 0.72	88.87 ± 0.75	89.34 ± 0.71	88.17 ± 0.76	6.54 ± 0.49	8.96 ± 0.63
Proposed method	92.16 ± 0.47	92.43 ± 0.51	91.68 ± 0.54	92.04 ± 0.49	90.86 ± 0.53	4.83 ± 0.34	6.74 ± 0.42

**Table 5:** Recognition performance of the proposed method under different risk states.

Risk State	Precision (%)	Recall (%)	F1-Score (%)	AUC (%)
Normal	94.28 ± 0.46	95.17 ± 0.43	94.72 ± 0.41	97.36 ± 0.35
Fatigue	91.36 ± 0.58	89.84 ± 0.63	90.59 ± 0.56	95.12 ± 0.44
Stress	90.74 ± 0.61	89.21 ± 0.66	89.97 ± 0.59	94.63 ± 0.47
Abnormal operation	93.15 ± 0.49	92.47 ± 0.52	92.81 ± 0.46	96.48 ± 0.38

**Table 6:** Normalized confusion matrix of the proposed method under different risk states.

Actual\Predicted	Normal	Fatigue	Stress	Abnormal Operation
Normal	95.17	1.84	1.29	1.70
Fatigue	2.16	89.84	5.73	2.27
Stress	1.71	6.12	89.21	2.96
Abnormal operation	1.48	2.13	3.92	92.47

**Table 7:** Ablation results of different components in the proposed method.

Model Variant	Accuracy (%)	Precision (%)	Recall (%)	F1-Score (%)	Macro-F1 (%)	FAR (%)	MDR (%)
w/o Physiological modality	79.84 ± 1.22	80.57 ± 1.25	78.96 ± 1.30	79.53 ± 1.26	78.16 ± 1.31	11.24 ± 0.89	17.58 ± 1.21
w/o Behavioral modality	80.92 ± 1.18	81.46 ± 1.21	80.08 ± 1.25	80.67 ± 1.20	79.28 ± 1.27	10.46 ± 0.86	16.27 ± 1.16
w/o Coupling module	88.21 ± 0.74	88.56 ± 0.77	87.42 ± 0.81	87.98 ± 0.76	86.73 ± 0.82	7.18 ± 0.55	9.84 ± 0.69
w/o Attention module	89.03 ± 0.70	89.38 ± 0.74	88.26 ± 0.77	88.79 ± 0.72	87.56 ± 0.79	6.83 ± 0.52	9.21 ± 0.66
w/o Temporal modeling	87.46 ± 0.79	87.81 ± 0.82	86.67 ± 0.86	87.22 ± 0.81	85.94 ± 0.88	7.61 ± 0.58	10.37 ± 0.73
Full model	92.16 ± 0.47	92.43 ± 0.51	91.68 ± 0.54	92.04 ± 0.49	90.86 ± 0.53	4.83 ± 0.34	6.74 ± 0.42

**Table 8:** Attention weights of physiological and behavioral modalities under different risk states.

Risk State	Physiological Modality	Behavioral Modality
Normal	0.48 ± 0.04	0.52 ± 0.04
Fatigue	0.61 ± 0.05	0.39 ± 0.05
Stress	0.66 ± 0.06	0.34 ± 0.06
Abnormal operation	0.37 ± 0.05	0.63 ± 0.05

## Data Availability

The datasets generated and analyzed during the current study are owned and managed by the relevant local government authorities. Due to data sensitivity and confidentiality agreements, the datasets are not publicly available.
